# Deep Visual Proteomics defines single-cell identity and heterogeneity

**DOI:** 10.1038/s41587-022-01302-5

**Published:** 2022-05-19

**Authors:** Andreas Mund, Fabian Coscia, András Kriston, Réka Hollandi, Ferenc Kovács, Andreas-David Brunner, Ede Migh, Lisa Schweizer, Alberto Santos, Michael Bzorek, Soraya Naimy, Lise Mette Rahbek-Gjerdrum, Beatrice Dyring-Andersen, Jutta Bulkescher, Claudia Lukas, Mark Adam Eckert, Ernst Lengyel, Christian Gnann, Emma Lundberg, Peter Horvath, Matthias Mann

**Affiliations:** 1 Proteomics Program, Novo Nordisk Foundation Center for Protein Research, Faculty of Health and Medical Sciences, University of Copenhagen, Copenhagen, Denmark; 2 Spatial Proteomics Group, Max Delbrück Center for Molecular Medicine in the Helmholtz Association, Berlin, Germany; 3 Synthetic and Systems Biology Unit, Biological Research Centre, Eötvös Loránd Research Network, Szeged, Hungary; 4 Single-Cell Technologies Ltd., Szeged, Hungary; 5 Proteomics and Signal Transduction, Max Planck Institute of Biochemistry, Martinsried, Germany; 6 Center for Health Data Science, University of Copenhagen, Copenhagen, Denmark; 7 Big Data Institute, Li-Ka Shing Centre for Health Information and Discovery, University of Oxford, Oxford, UK; 8 Department of Pathology, Zealand University Hospital, Roskilde, Denmark; 9 Institute for Clinical Medicine, University of Copenhagen, Copenhagen, Denmark; 10 Department of Dermatology and Allergy, Herlev and Gentofte Hospital, University of Copenhagen, Hellerup, Denmark; 11 Leo Foundation Skin Immunology Research Center, Faculty of Health and Medical Sciences, University of Copenhagen, Copenhagen, Denmark; 12 Protein Imaging Platform, Novo Nordisk Foundation Center for Protein Research, Faculty of Health and Medical Sciences, University of Copenhagen, Copenhagen, Denmark; 13 Protein Signaling Program, Novo Nordisk Foundation Center for Protein Research, Faculty of Health and Medical Sciences, University of Copenhagen, Copenhagen, Denmark; 14 Department of Obstetrics and Gynecology/Section of Gynecologic Oncology, University of Chicago, Chicago, IL USA; 15 Science for Life Laboratory, School of Engineering Sciences in Chemistry, Biotechnology and Health, KTH - Royal Institute of Technology, Stockholm, Sweden; 16 Department of Bioengineering, Stanford University, Stanford, CA USA; 17 Chan Zuckerberg Biohub, San Francisco, CA USA; 18 Institute for Molecular Medicine Finland (FIMM), University of Helsinki, Helsinki, Finland

**Keywords:** Proteomics, Mechanisms of disease, Tumour heterogeneity, Single-cell imaging

## Abstract

Despite the availabilty of imaging-based and mass-spectrometry-based methods for spatial proteomics, a key challenge remains connecting images with single-cell-resolution protein abundance measurements. Here, we introduce Deep Visual Proteomics (DVP), which combines artificial-intelligence-driven image analysis of cellular phenotypes with automated single-cell or single-nucleus laser microdissection and ultra-high-sensitivity mass spectrometry. DVP links protein abundance to complex cellular or subcellular phenotypes while preserving spatial context. By individually excising nuclei from cell culture, we classified distinct cell states with proteomic profiles defined by known and uncharacterized proteins. In an archived primary melanoma tissue, DVP identified spatially resolved proteome changes as normal melanocytes transition to fully invasive melanoma, revealing pathways that change in a spatial manner as cancer progresses, such as mRNA splicing dysregulation in metastatic vertical growth that coincides with reduced interferon signaling and antigen presentation. The ability of DVP to retain precise spatial proteomic information in the tissue context has implications for the molecular profiling of clinical samples.

## Abstract (web summary)

Deep Visual Proteomics combines machine learning, automated image analysis and single-cell proteomics.

## Main

**Fig. 1:**
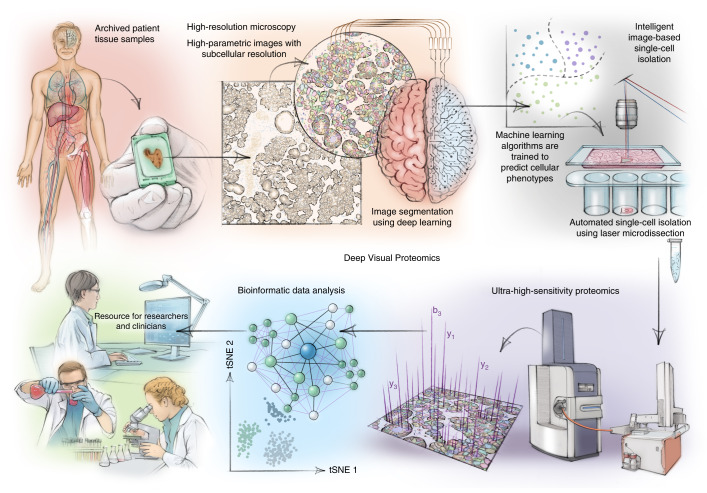
DVP concept and workflow. DVP combines high-resolution imaging, AI-guided image analysis for single-cell classification and isolation with an ultra-sensitive proteomics workflow^2^. DVP links data-rich imaging of cell culture or archived patient biobank tissues with deep-learning-based cell segmentation and machine-learning-based identification of cell types and states. (Un)supervised AI-classified cellular or subcellular objects of interest undergo automated LMD and MS-based proteomic profiling. Subsequent bioinformatics data analysis enables data mining to discover protein signatures, providing molecular insights into proteome variation in health and disease states at the level of single cells. tSNE, t-distributed stochastic neighbor embedding.

Modern microscopyʼs versatility, resolution and multi-modal nature delivers increasingly detailed images of single-cell heterogeneity and tissue organization^1^. Currently, a predefined subset of proteins is usually targeted, far short of the actual complexity of the proteome. Taking advantage of substantially increased sensitivity in technology based on mass spectrometry (MS), we set out to enable the analysis of proteomes within their native, subcellular context to explore their contribution to health and disease. We combined sub-micron-resolution imaging, image analysis for single-cell phenotyping based on artificial intelligence (AI) and isolation with an ultra-sensitive proteomics workflow^2^ (Fig. 1). Key challenges turned out to be the accurate definition of single-cell boundaries and cell classes as well as the transfer of the automatically defined features into proteomic samples, ready for analysis. To this end, we introduce the software ‘BIAS’ (Biology Image Analysis Software), which coordinates scanning and laser microdissection (LMD) microscopes. This seamlessly combines data-rich imaging of cell cultures or archived biobank tissues (formalin-fixed and paraffin-embedded (FFPE)) with deep-learning-based cell segmentation and machine-learning-based identification of cell types and states. Cellular or subcellular objects of interest are selected by the AI alone or after instruction before being subjected to automated LMD and proteomic profiling. Data generated by DVP can be mined to discover protein signatures providing molecular insights into proteome variation at the phenotypic level while retaining complete spatial information.

## Results

### Image-guided single-cell isolation for cell-type-resolved proteomics

The microscopy-related aspects of the DVP workflow build on high-resolution whole-slide imaging, machine learning (ML) and deep learning (DL) for image analysis.

**Fig. 2:**
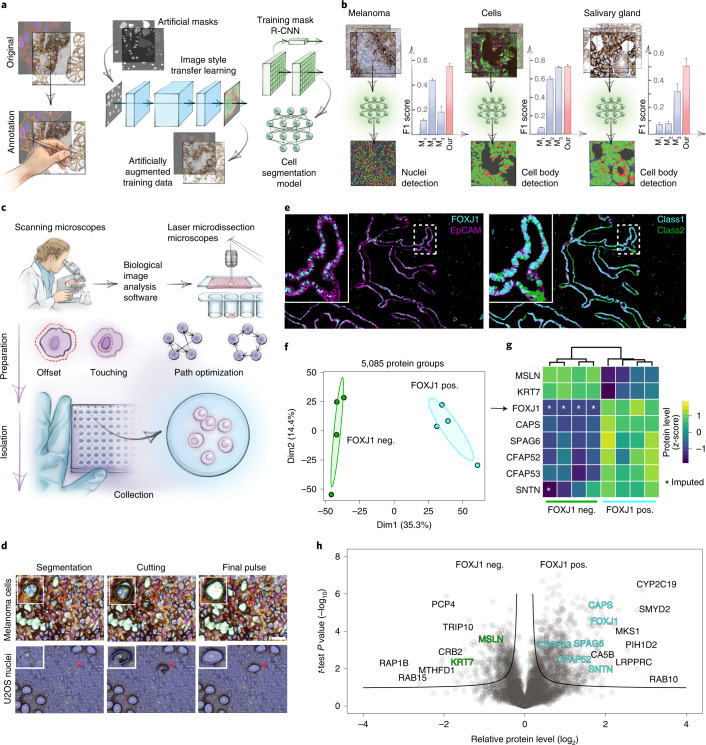
BIAS for integrative image analysis and automated LMD single-cell isolation. **a**, AI-driven nucleus and cytoplasm segmentation of normal-appearing and cancer cells and tissue using BIAS. **b**, We benchmarked the accuracy of its segmentation approach using the F1 metric and compared results to three additional methods—M_1_ is unet4nuclei^6^, M_2_ is CellProfiler^8^ and M_3_ is Cellpose^7^—while OUR refers to nucleAIzer^3^. Bars show mean F1 scores with s.e.m.; *n* = 10 independent images for melanoma tissue and (U2OS) cells, and *n* = 20 for salivary gland tissue. Visual representation of the segmentation results: green areas correspond to true positive, blue to false positive and red to false negative. **c**, BIAS serves as the interface between the scanning and an LMD microscope, allowing high-accuracy transfers of cell contours between the microscopes. Illustration of cutting offset with respect to the object of interest and optimal path finding. **d**, Practical illustration of the functions in the upper panel. **e**, Immunofluorescence staining of the human fallopian tube epithelium with FOXJ1 and EpCAM antibodies, detecting ciliated and epithelial cells, respectively. Left panel: Ciliated (FOXJ1-positive) and secretory (FOXJ1-negative) cells. Right panel: Cell classification based on FOXJ1 intensity. Class 1 (FOXJ1-positive) and class 2 (FOXJ1-negative); magnification factor = ×387. **f**, PCA of FOXJ1-positive and FOXJ1-negative cell proteomes. **g**, Heat map of known protein markers for secretory and ciliated cells. Protein levels are z-scored. Asterisks represent imputed data. The marker list was derived from the Human Protein Atlas^20^ project and based on literature mining. **h**, Volcano plot of the pairwise proteomic comparison between FOXJ1-positive and FOXJ1-negative cells. Cell-type-specific marker proteins are highlighted in green and turquoise, and black represents potential novel marker proteins. Significant enriched cell-type-specific proteins are displayed above the black lines (two-sided *t*-test, FDR < 0.05, *s*_*0*_ = 0.1, *n* = 4 biological replicates).

**Extended Data Fig. 1:**
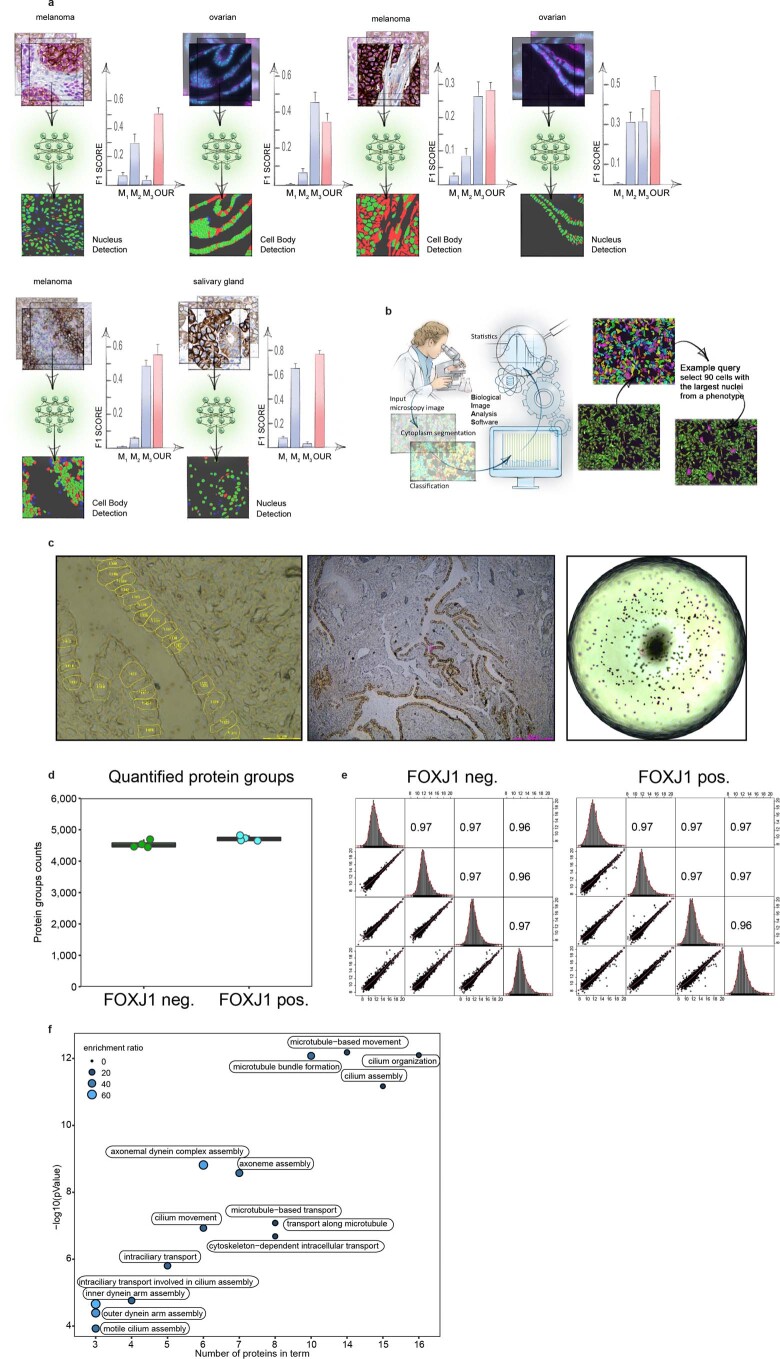
Benchmarking of segmentation algorithm. **a**, Cell body and nuclei segmentation of melanoma, salivary gland and fallopian tube tissue using the Biological Image Analysis Software (BIAS). We benchmarked the accuracy of our segmentation approach using the F1 metric and compared results to three additional methods M1-M3. unet4nuclei (M_1_)^6^, CellProfiler (M2)^8^, CellPose (M3)^7^, while OUR refers to nucleAIzer^3^. Bars show mean F1-scores with SEM (standard error of the mean). Visual representation of the segmentation results: green areas correspond to true positive, blue to false positive and red to false negative. Data provided in Table 1 and Supplementary Table 1. **b**, BIAS allows the processing of multiple 2D and 3D microscopy image file formats. Examples for image pre-processing, deep learning-based image segmentation, feature extraction and machine learning-based phenotype classification. **c**, Left: Contour alignment in the LMD7 software before laser microdissection of fallopian tube epithelial cells. Middle: Screenshot after laser microdissection. Right: 384-well inspection after laser microdissection in individual fallopian tube epithelial cells. **d**, Number of quantified proteins per replicate of FOXJ1 positive and negative epithelial cells. Samples were acquired in data-independent mode and analyzed with the DIA-NN software. **e**, Replicate correlations of proteome measurements. Correlation values show Pearson correlations. **f**, Pathway enrichment analysis for proteins significantly higher in ciliated cells compared to secretory fallopian tube epithelial cells.

First, we used scanning microscopy to obtain high-resolution whole-slide images and developed a software suite for integrative image analysis termed ‘BIAS’ (Methods). BIAS processes multiple two-dimensional (2D) and three-dimensional (3D) microscopy image file formats, supporting major microscope vendors and data formats. It combines image pre-processing, DL-based image segmentation, feature extraction and ML-based phenotype classification. Building on a recent DL-based algorithm for cytoplasm and nucleus segmentation^3^, we undertook several optimizations to implement pre-processing algorithms to maintain high-quality images across large image datasets. DL methods require large training datasets, which is a considerable challenge due to the limited size of high-quality training data^4^. To address this challenge, we used nucleAIzer^3^ and applied project-specific image style transfer to synthesize artificial microscopy images resembling real images. This approach is inherently adaptable to different biological scenarios, such as new cell and tissue types or staining techniques^5^. We trained a deep neural network with these synthetic images for specific segmentation of the cellular compartment of interest (for example, nucleus or cytoplasm; Fig. 2a). We benchmarked it against two leading DL approaches—unet4nuclei^6^ and Cellpose^7^—and a widely used adaptive threshold-based and object-splitting-based method^8^. Our cell and nucleus segmentation algorithms of cell cultures and tissues showed the highest accuracy (Fig. 2b, Extended Data Fig. 1a, Table 1 and Supplementary Table 1). Our current benchmarking results are supported by a previous study^3^ where we performed an extensive comparison to additional methods and software (for example, ilastik^9^, on a large heterogeneous microscopy image set). For interactive cellular phenotype discovery, BIAS performs phenotypic feature extraction, taking into account morphology and neighborhood features based on supervised and unsupervised ML (Extended Data Fig. 1b and Methods). Feature-based phenotypic classification is readily combined with biomarker expression level from antibody staining for precise cell classification. ML has previously been used for image analysis and cell selection but not combined with unbiased proteomics^10^. Furthermore, we extended BIAS with a Python interface; thus, data access and manipulation is also possible using standard Python functions in a generic way, including the integration of open-source packages and custom algorithms.

**Table 1:** Mean F1 scores of the compared segmentation methods on our samples

Sample	Method			
	M_1_	M_2_	M_3_	OUR
U2OS cyto	0.0667* ± 0.0075	0.5994 ± 0.0262	0.7205 ± 0.0152	**0.7336** ± 0.0218
Melanoma nuc	0.1126 ± 0.0151	0.4386 ± 0.0157	0.1801 ± 0.0504	**0.5498** ± 0.0231
Melanoma cyto	0.0058* ± 0.0021	0.0549 ± 0.0083	0.4859 ± 0.0354	**0.5536** ± 0.0625
Salivary gland nuc	0.0797 ± 0.0138	0.6488 ± 0.0430	0.0338 ± 0.0145	**0.7684** ± 0.0316
Salivary gland cyto	0.0714* ± 0.0151	0.0793 ± 0.0167	0.3174 ± 0.0588	**0.5051** ± 0.0586
Melanoma (pink) nuc	0.0682 ± 0.0183	0.2999 ± 0.0599	0.0364 ± 0.0238	**0.5079** ± 0.0392
Melanoma (pink) cyto	0.0261* ± 0.0070	0.0865 ± 0.0213	0.2659 ± 0.0429	**0.2839** ± 0.0229
Fallopian tube nuc	0.0006 ± 0.0009	0.3121 ± 0.0501	0.3160 ± 0.0631	**0.4724** ± 0.0683
Fallopian tube cyto	0.0016* ± 0.0023	0.0671 ± 0.0208	**0.4566** ± 0.0530	0.3455 ± 0.0473

The methods are as follows: M_1_ is unet4nuclei^6^, M_2_ is CellProfiler^8^, M_3_ is Cellpose^7^ and OUR refers to nucleAIzer^3^ (implemented in BIAS). High scores are highlighted in bold. Asterisks (*) mark that M_1_ is intended for nucleus segmentation but was applied to segment cytoplasm. s.e.m. is displayed with ± after the mean F1 scores in each cell.

To physically extract the cellular features discovered with BIAS, we developed an interface between scanning and LMD microscopes (currently Zeiss PALM MicroBeam and Leica LMD6 and LMD7) (Fig. 2c). BIAS transfers cell contours between the microscopes, preserving full accuracy. LMD has a theoretical accuracy of 70 nm using a ×150 objective, but, in practice, we reached 200 nm. After optimization, the LMD7 can autonomously excise 1,250 high-resolution contours per hour, equivalent to 50 to 100 cells per sample (Methods). To prevent potential laser-induced damage to cell membranes, we excise contours with an offset (Fig. 2c,d and Supplementary Videos 1 and 2).

Current LMD methods preserve the spatial context but are mostly limited to human-eye-observable phenotypes and require manual selection of cells, often resulting in admixing of different cell types, which constrains throughput and de novo discovery^11^.

To explore the sensitivity, specificity and robustness of our DVP workflow, we obtained normal human fallopian tube tissue and separated ciliated from secretory cells—the two major cell types of the fallopian tube epithelium^12^—using the cell-lineage-specific transcription factor FOXJ1, a master regulator of cilia function, and measured their proteomes (Fig. 2e–h, Extended Data Fig. 1c–f and Supplementary Table 2). We solely detected FOXJ1 (ciliated cells) in FOXJ1-stained cells (Fig. 2e,g), along with more than 5,000 other quantified proteins with excellent correlations of biological replicates (Extended Data Fig. 1d,e). Bioinformatic analysis of differences in protein abundance mirrored the biologic features of the distinct cell types. (Fig. 2f–h and Extended Data Fig. 1c–f). This was driven by known protein markers of ciliated cells and expanded to proteins not yet functionally associated with these cell types. We used the fallopian tube epithelium as an example to highlight the importance of the combination of antibody-based tissue staining and unbiased, quantitative proteomics. Such in vivo cell type comparisons will allow the discovery of cell type and cell state markers and provide unbiased information to understand disease states at the global proteome level. Of note, high-grade serous ovarian cancer originates in the fallopian tube epithelium, and our method can now be applied to study the early onset of the disease without admixing unrelated cell types^13^.

### DVP defines single-cell heterogeneity at the subcellular level

**Fig. 3:**
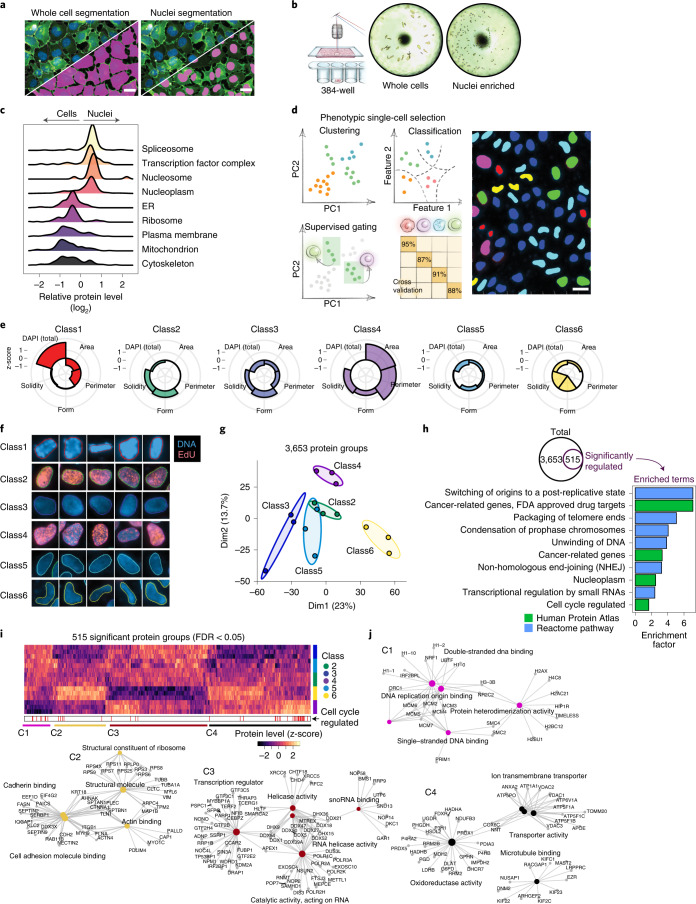
DVP defines single-cell heterogeneity at the subcellular level. **a**, Segmentation of whole cells and nuclei in BIAS of DNA (DAPI)-stained U2OS cells. Scale bar, 20 μm **b**, Automated LMD of whole cells and nuclei into 384-well plates. Images show wells after collection. **c**, Relative protein levels (*x* axis) of major cellular compartments between whole cell (*﻿n* = 3 biological replicates) and nuclei (*﻿n* = 3 biological replicates) specific proteomes. *y* axis displays point density. **d**, Left: conceptual workflows of the phenotype finder model of BIAS for ML-based classification of cellular phenotypes. Right: results of unsupervised ML-based classification of six distinct U2OS nuclei classes based on morphological features and DNA staining intensity. Colors represent classes. Scale bar, 20 μm. **e**, Phenotypic features used by ML to define six distinct nuclei classes. Radar plots show z-scored relative levels of morphological features (nuclear area, perimeter, solidity and form factor) and DNA staining intensity (total DAPI signal). **f**, Example images of nuclei from the six classes identified by ML. Blue color shows DNA staining intensity, and red color shows EdU staining intensity to identify cells undergoing replication. Represented nuclei are enlarged for visualization and do not reflect actual sizes. **g**, PCA of five interphase classes based on 3,653 protein groups after data filtering. Replicates of classes (*﻿n* = 3 biological replicates) are highlighted by ellipses with a 95% confidence interval. **h**, Enrichment analysis of proteins regulated among the five nuclei classes. Significant proteins (515 ANOVA significant, FDR < 0.05, *s*_*0*_ = 0.1) were compared to the set of unchanged proteins based on Gene Ontology Biological Process (GOBP), Reactome pathways as well as cell cycle and cancer annotations derived from the Human Protein Atlas (HPA)^20^. A Fisher’s exact test with a Benjamini–Hochberg FDR of 0.05 was used (Supplementary Table 3). **i**, Unsupervised hierarchical clustering of all 515 ANOVA significant protein groups (Supplementary Table 4). Cell-cycle-regulated proteins reported by the HPA are shown in the lower bar. Nuclei classes (*﻿n* = 3 biological replicates) are shown in the row bar. C1–C4 show clusters upregulated in the different nucleus classes. **j**, Network analysis of enriched pathways for protein clusters C1–C4. Pathway enrichment analysis was performed with the ClusterProfiler R package^36^. ER, endoplasmic reticulum; PC, principal component.

**Extended Data Fig. 2:**
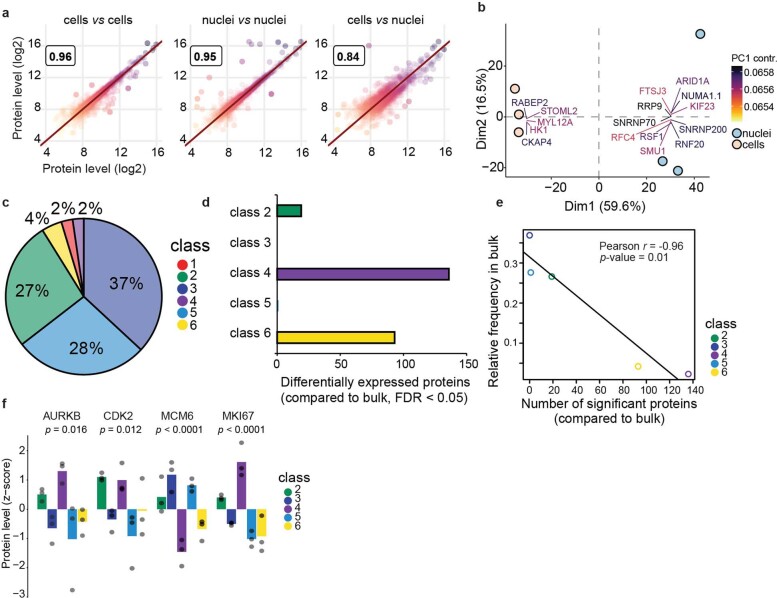
PCA and loadings of cell culture classes at sub-cellular level and number of significantly changed proteins vs. class abundance. **a**, Quantitative proteomic results of whole cell and nuclei replicates, and comparison between whole cells and nuclei. **b**, Principal component analysis (PCA) of whole cell (*n* = 3) and nuclei proteomes (*n* = 3). Proteins with the strongest contribution to PC1 are highlighted. **c**, Relative proportions of the six nuclei classes. **d**, Number of differentially expressed proteins (two-sided *t*-test, *n* = 3 biological replicates) compared to unclassified nuclei (bulk). Proteins with an FDR less than 0.05 were considered significant. **e**, Correlation between number of significantly regulated proteins per nuclei class vs relative class proportion. A linear model was fitted to the data showing an inverse correlation with Pearson *r* = -0.96 (*p*-value = 0.01). **f**, Relative protein levels (z-score) of known cell cycle markers across the five nuclei classes. All bar graphs represent mean of data (*n* = 3 biological replicates) and error bars are s.d. ANOVA *p*-values are shown.

We applied our workflow to an unperturbed cancer cell line to determine if DVP can characterize functional heterogeneity between ostensibly similar cells (fluorescent ubiquitination-based cell cycle indicator (FUCCI) U2OS cells^14^). After DL-based segmentation for nuclei and cell membrane detection, we isolated 80–100 single cells or 250–300 nuclei per phenotype (Figs. 2c,d and 3a,b). The analysis of small numbers of cells by MS has been a longstanding goal, held back by formidable analytical challenges in the transfer, processing and analysis of minute samples^15^, which we addressed in turn. We processed samples using our recently developed workflow for ultra-low sample input^2,16^, which omits any sample transfer steps and ensures de-crosslinking in very low volumes (Methods). We found that samples could be analyzed directly from 384 wells without any additional sample transfer or clean-up. For MS measurements, we employed a data-independent acquisition method using parallel accumulation–serial fragmentation with an additional ion mobility dimension and optimal fragment (diaPASEF) ion recovery on a newly developed mass spectrometer^2,17^. Replicates of cell and nucleus proteomes demonstrated high quantitative reproducibility (Pearson *r* = 0.96), and proteomes of whole cells differed from those of nuclei alone, as expected from subcellular proteomics experiments based on biochemical separation^18^ (Extended Data Fig. 2a,b). In the bioinformatic enrichment analysis, terms like plasma membrane, mitochondrion, nucleosomes and transcription factor complexes were highly significant (false discovery rate (FDR) < 10^−5^) (Fig. 3c).

**Extended Data Fig. 3:**
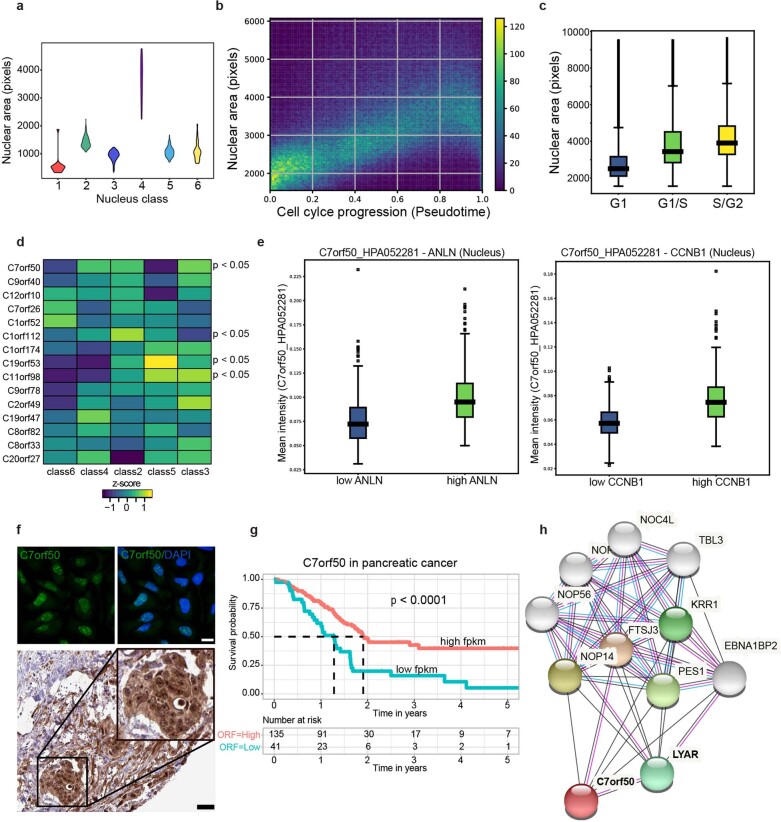
DVP discovers uncharacterized proteins with potential clinical relevance. **a**, Violin plots showing nuclear area in pixels of the 6 nuclei classes identified by ML. **b**, Nuclear area in pixels of U2OS FUCCI cells in relation to the cell cycle pseudotime^14^. Color code indicates point density. **c**, Nuclear area of three major cell cycle states G1, G1/S and S/G2 determined by fluorescently tagged CDT1 and GMNN intensities and Gaussian clustering. Box plots show the results of *n* = 238,675 cells in total (85,551 for G1, 83,121 for G1/S and 70,003 for S/G2). **d**, Relative protein levels of all identified ORF proteins in the dataset. C7orf50, C1orf112, C19orf53 and C11orf98 were differentially expressed (ANOVA *p*-value < 0.05) across the 5 nuclei classes (*n* = 3 biological replicates). **e**, Mean intensities of immunofluorescent stained C7orf50 and the cell cycle markers ANLN and CCNB1 in U20S cells. C7orf50 levels were quantified in nuclei with low and high ANLN and CNNB1 intensities. Box plots show the results of *n* = 263 cells per condition (C7orf50-ANLN) and n = 412 per condition (C7orf50-CCNB1). **f**, Upper panel: Representative immunofluorescence images of C7orf50 and DNA (DAPI) stained U2OS cells^19^. Scale bar is 20 µm. Note, C7orf50 is enriched in nucleoli. Lower panel: Immunohistochemistry of a C7orf50 stained pancreatic adenocarcinoma (https://bit.ly/2X4re05). Image credit: Human Protein Atlas. Scale bar is 40µm. **g**, Kaplan-Meier survival analysis of pancreatic adenocarcinoma (https://bit.ly/3BAxewA) based on relative C7orf50 RNA levels (FPKM, number of Fragments Per Kilobase of exon per Million reads). RNA-seq data is reported as median FPKM, generated by The Cancer Genome Atlas (https://bit.ly/3iSOG8d). Patients were divided into two groups based on C7orf50 levels with n=41 low and n=135 high patients. A log-rank test was calculated with *p* = 0.0001. **h**, String interactome analysis for C7orf50. A high confidence score of 0.7 was used with the five closest interactors highlighted by color^54^. The box plots in c and e define the range of the data (whiskers), 25th and 75th percentiles (box), and medians (solid line). Outliers are plotted as individual dots outside the whiskers.

To address if morphological differences between nuclei are also reflected in their proteomes, we used an unsupervised phenotype finder model to identify groups of morphologically distinct nuclei based on nuclear area, perimeter, form factor, solidity and DNA staining intensity (Fig. 3d). ML found three primary nuclei classes (27–37% each) and also identified three rare ones (2–4% each) (Extended Data Fig. 2c). The resulting six distinct nuclei classes had visible differences in size and shape, with class 1 representing mitotic states and the remaining five classes representing interphase with varying feature weighting (Fig. 3e,f). We focused on those five nuclei classes of unknown origin for subsequent analysis. In principal component analysis (PCA), replicates of the respective proteomes clustered closely, and the more frequent classes (2, 3 and 5) grouped together (Fig. 3g). To verify and quantify this observation, we compared each cell class proteome to a proteome of all ‘mixed’ nuclei in a field of view. This revealed that the rarest cell classes had the highest numbers of differentially expressed proteins compared to unclassified ‘bulk’ proteomes (Extended Data Fig. 2d,e). We next asked if the proteomic differences across the five nuclei classes suggested any functional differences among the interphase states (Fig. 3d,f). The 515 significantly differentially expressed proteins across classes were enriched for nuclear and cell-cycle-related proteins (for example, ‘switching of origins to a post-replicative state’ and ‘condensation of prophase chromosomes’), suggesting the cell cycle as a functional driver of separation (Fig. 3h–j, Extended Data Fig. 2f and Supplementary Tables 3 and 4). Comparing our data to a single-cell imaging dataset of cell-cycle-regulated proteins^19^, we found significant enrichment in our regulated proteins (FDR < 10^−6^). Nuclear area, one of the driving features among the different classes identified, increased during interphase from G1 to S/G2 cells (Fig. 3e and Extended Data Fig. 3a–c), further supporting the importance of the cell cycle in defining the nuclei classes.

Our single-cell-type proteomes discovered several uncharacterized proteins, presenting an opportunity to associate them with a potential cellular function. Focusing on C11orf98, C7orf50, C1orf112 and C19orf53, which remained after data filtering (ANOVA *P* <0.05), showed class-specific expression patterns (Extended Data Fig. 3d). C7orf50 was most highly expressed in the nucleoli of classes 2, 4 and 3 nuclei, which showed S/G2-specific characteristics (Fig. 3f and Extended Data Fig. 3d,e), suggesting that its expression is cell cycle regulated. Indeed, we confirmed higher levels of C7orf50 in G1/S and S/G2 compared to G1 phase cells (Extended Data Fig. 3e). As cell-cycle-regulated proteins may be associated with cancer prognosis^19^, we investigated C7orf50 in the human pathology atlas^20^ where high expression was associated with favorable outcomes in pancreatic cancer (Extended Data Fig. 3g; *P* < 0.001). Bioinformatic analysis revealed interaction, co-expression and co-localization with the protein LYAR (‘cell growth-regulating nucleolar protein’), suggesting a functional link to cell proliferation (Extended Data Fig. 3f,h).

Class 6 showed an intriguing proteomic signature independent of known cell cycle markers (Fig. 3i,j). These rare, bean-shaped nuclei showed upregulation of specific cytoskeletal and cell adhesion proteins (for example, VIM, TUBB, ACTB and ITGB1), suggesting that these signatures derived from migrating cells undergoing nuclear deformation, suggestive of cellular invasion^21,22^. Note that we classified nuclei from 2D images, but LMD isolates them in 3D. Thus, samples also probe morphology-driven protein re-localization around the nucleus as exemplified by class 6 nuclei. Likewise, excising the nuclei captures the trafficking of proteins to and from the cytosol to some degree.

These cell culture experiments establish that DVP correlates cellular phenotypes, heterogeneity and dynamics with the proteome level in an unbiased way for common and rare phenotypes.

### DVP applied to cancer tissue heterogeneity

**Fig. 4:**
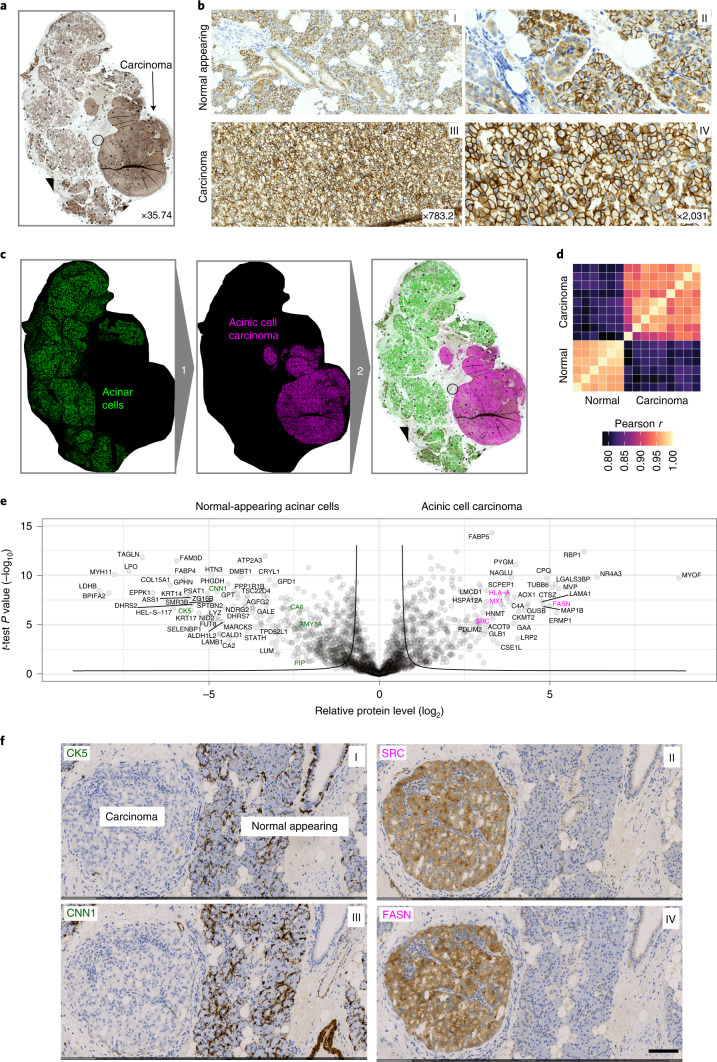
DVP applied to archived tissue of a rare salivary gland carcinoma. **a**, IHC staining of an acinic cell carcinoma of the salivary gland using the cell adhesion protein EpCAM. **b**, Representative regions from normal-appearing tissue (upper panels I and II) and acinic cell carcinoma (lower panels III and IV) from **a**. **c**, DVP workflow applied to the acinic cell carcinoma tissue. DL-based single cell detection of normal-appearing (green) and neoplastic (magenta) cells positive for EpCAM. Cell classification based on phenotypic features (form factor, area, solidity, perimeter and EpCAM intensity). **d**, Proteome correlations of replicates from normal-appearing (normal, *n* = 6) or cancer regions (cancer, *n* = 9). **e**, Volcano plot of pairwise proteomic comparison between normal and cancer tissue. *t*-test significant proteins (two-sided *t*-test, FDR < 0.05, *s*_*0*_ = 0.1, *﻿n* = 6 biological replicates for normal and *n* = 9 for cancer) are highlighted by black lines. Proteins more highly expressed in normal tissue are highlighted in green on the volcanoʼs left, including known acinic cell markers (AMY1A, CA6 and PIP). Proteins more highly expressed in the acinic cell carcinoma are on the right in magenta, including the proto-oncogene SRC and interferon response proteins (MX1 and HLA-A; Supplementary Table 6). **f**, IHC validation of proteomic results. CNN1, SRC, CK5 and FASN are significantly enriched in normal or cancer tissue. Scale bar, 100 μm.

Billions of patient samples are collected routinely during diagnostic workup and stored in the archives of pathology departments around the world^23^. The precise proteomic characterization of single cells in their spatial and subcellular context from tissue slides could have a tremendous clinical effect, complementing the emerging field of digital pathology^24^. We selected archived paraffin-embedded tissue of a salivary gland acinic cell carcinoma, a rare and understudied malignancy of epithelial secretory cells of the salivary gland. We developed an immunohistochemical (IHC) staining protocol on glass membrane slides for LMD and stained the tissue for EpCAM to outline the cellular boundaries for segmentation and feature extraction by BIAS (Methods). These histologically normal-appearing regions were mainly comprised of acinar, ductal and myoepithelial cells, whereas the carcinoma component had predominatly uniform tumor cells with round nuclei and abundant basophilic cytoplasm (Fig. 4a,b).

**Extended Data Fig. 4:**
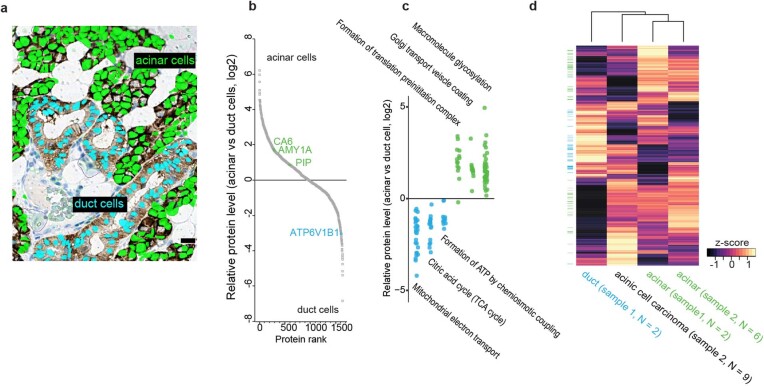
DVP applied to archival tissue of a rare salivary gland carcinoma. **a**, Immunohistochemical staining of normal salivary gland stained for the cell adhesion protein EpCAM. Supervised (random forest) ML was trained to identify acinar (green) and duct cells (turquoise). Scale bar = 20µm. **b**, Quantitative proteomic comparison between acinar and duct cells from tissue in A with known cell type specific markers highlighted (https://bit.ly/3iOK8Qf). **c**, Relative protein levels of selected pathways that were significantly higher in acinar or duct cells. **d**, Unsupervised hierarchical clustering of acinar and duct cell proteomes from two different patients together with acinar cell carcinoma cells. Note that normal acinar cells of two different tissues clustered together. Duct cells clustered furthest away. Prior to clustering, protein levels from different sample groups (duct cell tissue #1, acinar cell tissue #1, acinar cell tissue #2, carcinoma tissue #2) were averaged and z-scored. Bar on the left shows differentially expressed pathways from panel b with acini and duct specific proteins in green and turquoise, respectively.

To identify disease-specific protein signatures, we aimed to compare the histologically normal-appearing acinar cells with the malignant cells rather than admixing with varying proportions of unrelated cells. To this end, we classified acinar and duct cells from normal parotid gland tissue based on their cell-type-specific morphological features and isolated single-cell classes for proteomic analysis (Fig. 4c and Extended Data Fig. 4a). Bioinformatics analysis of the measured proteome differences revealed significant biological differences between these neighboring cell types, reflecting their distinct physiological functions. Acinar cells, which produce and secrete saliva in secretory granules, showed high expression of proteins related to vesicle transport and glycosylation along with known acinar cell markers such as α-amylase (AMY1A), CA6 and PIP (Extended Data Fig. 4b). In contrast, ductal cells expressed high levels of mitochondria and metabolism-related proteins required to meet the high energy demand for saliva secretion^25^ (Extended Data Fig. 4c and Supplementary Table 5). For comparison, we exclusively excised malignant and benign acinar cells from the various regions within the same tissue section. The proteomes of acinar cells clustered together regardless of disease state, indicating a strong cell-of-origin signature (Extended Data Fig. 4d). Analyzing six normal-appearing replicates and nine neoplastic regions showed excellent within-group proteome correlation (Pearson *r* > 0.96). The lower correlation of normal cells and cancer cells reflected disease-specific and cell-type-specific proteome changes (Pearson *r* = 0.8; Fig. 4d,e and Supplementary Table 6). Acinar cell markers in the carcinoma were significantly downregulated, consistent with previous reports^25^. DVP allowed us to discover upregulation of interferon response proteins (for example, MX1 and HLA-A; Supplementary Table 6) and the proto-oncogene SRC, both actionable therapeutic targets^26^ (Fig. 4e). We validated the proteomic findings using IHC analysis of significantly enriched proteins in either normal-appearing or cancererous tissue. This resulted in the selection of CNN1, SRC, CK5 and FASN (Fig. 4f), which confirmed our proteomic results, demonstrated the absence of contamination and supported the specificity of our DVP approach.

**Fig. 5:**
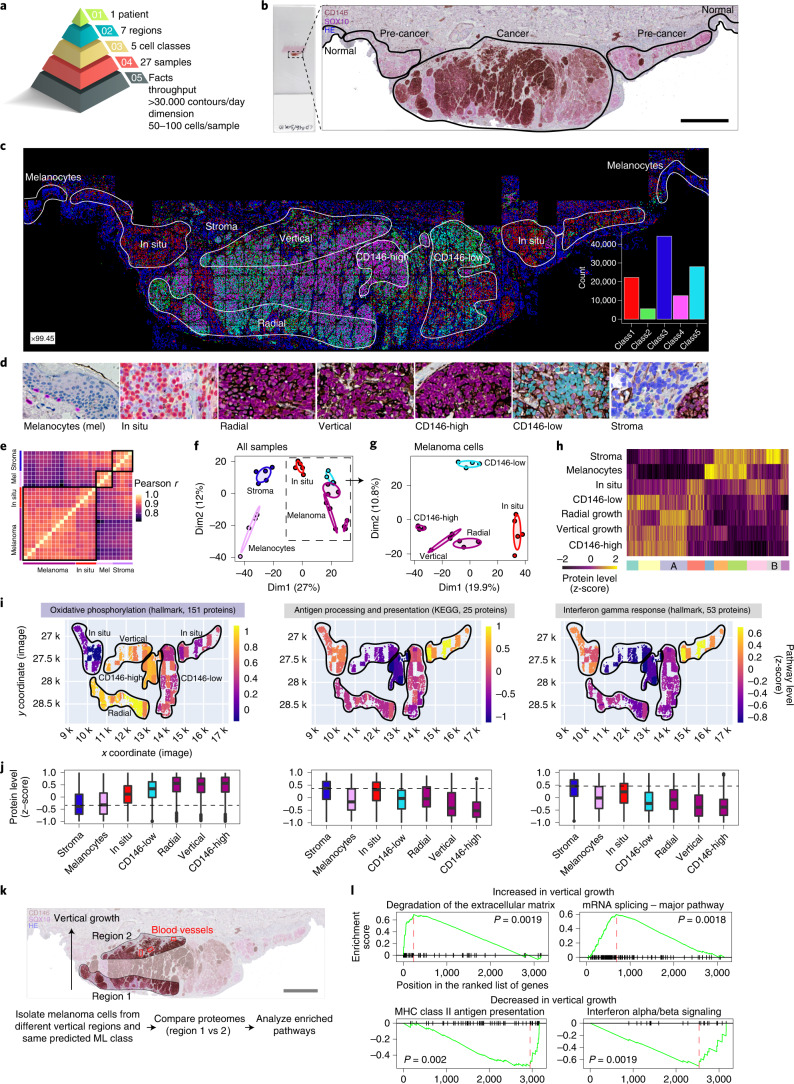
DVP applied to archived primary melanoma tissue. **a**, DVP sample isolation workflow to profile primary melanoma. **b**, DVP applied to primary melanoma immunohistochemically stained for the melanocyte marker SOX10 and the melanoma marker CD146. Left panel: stained melanoma tissue on a PEN glass membrane slide. Right panel: pathology-guided annotation of different tissue regions. Scale bar, 1 mm. **c**, Pathologist-guided and ML-based cell classification based on CD146 and SOX10 staining intensity and spatial localization: normal melanocytes, stromal cells, melanoma in situ, CD146-low melanoma, CD146-high melanoma, radial growth melanoma and vertical growth melanoma. Right lower panel: frequency of classes predicted by unsupervised ML (*k*-means clustering). **d**, Example pictures of the seven identified classes. Magnification factor = ×4,400. **e**, Correlation matrix (Pearson *r*) of all 27 measured proteome samples. **f**, PCA of proteomes. **g**, PCA of all melanoma-specific proteomes from in situ to invasive (vertical growth) melanoma. **h**, Unsupervised hierarchical clustering based on all 1,910 ANOVA significant (FDR < 0.05) protein groups. Two clusters of upregulated (cluster A) or downregulated (cluster B) proteins in invasive melanoma are highlighted. **i**, Tissue heat map mapping the proteomics results onto the imaging data. Relative pathway levels of selected terms from the two clusters are highlighted in **i**. Median protein levels were calculated per annotation and plotted for each isolated cell class against their *x* and *y* coordinates, as defined by their segmented cellular contours. **j**, Box plots of z-scored protein levels for the differentially regulated pathways visualized in **i** above. The box plots define the range of the data (whiskers), 25th and 75th percentiles (box) and medians (solid line). Outliers are plotted as individual dots outside the whiskers. **k**, Comparing proteomic changes in CD146-high melanoma cells (class 4) of the vertical growth (region 2) with the radial growth (region 1). Blood vessels in proximity to melanoma cells of the vertical growth are highlighted in red. Scale bar, 1 mm. **l**, Gene set enrichment analysis plot of significantly enriched pathways for melanoma cells of the vertical and radial growth phase. ﻿Pathway enrichment analysis was based on the protein fold change between vertical and radial melanoma cells and performed with the ClusterProfiler R package^36^. Enriched terms with an FDR < 0.05 are shown. MHC, major histocompatibility complex.

**Extended Data Fig. 5:**
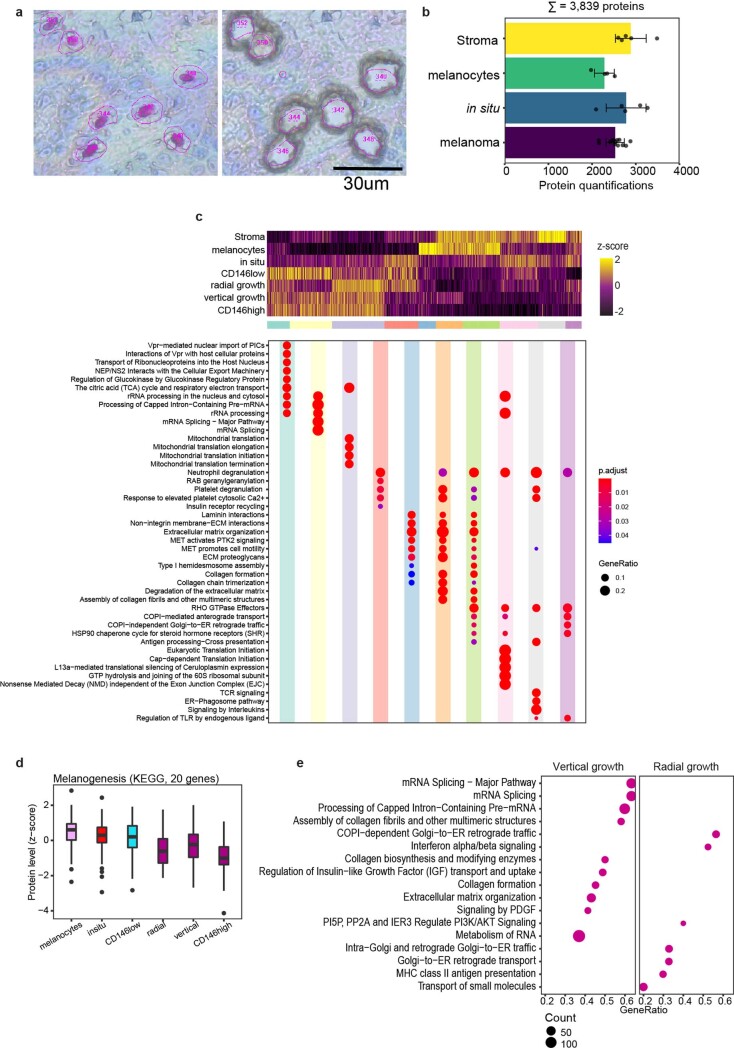
DVP applied to archival tissue of primary melanoma. **a**, Isolation of tumor adjacent SOX10 positive melanocytes from a cutaneous melanoma tissue. Left: Contour alignment before laser microdissection. Right: Inspection after laser microdissection. **b**, Number of protein quantifications per sample type with *n* = 4 (melanocytes), *n* = 5 (stroma), *n* = 5 (melanoma in situ) and *n* = 13 (melanoma) independent replicates. Bar graphs represent mean of data and error bars are s.d. Samples were acquired in data-independent mode and analyzed with the DIA-NN software. **c**, Upper panel: Heatmap from Fig. 5h shown with identified protein clusters (color bar). Unsupervised hierarchical clustering based on all 1,910 ANOVA significant (FDR < 0.05) protein groups. Protein levels were z-scored. Lower panel: Pathway enrichment analysis of different row clusters obtained by unsupervised hierarchical clustering. The ReactomePA package was used for enrichment analysis with an FDR cut-off of 0.05 for all enriched terms. **d**, Relative levels (z-score) of proteins related to the KEGG term ‘melanogenesis’. Note, melanocytes show highest protein levels. The box plots define the range of the data (whiskers), 25th and 75th percentiles (box), and medians (solid line). Outliers are plotted as individual dots outside the whiskers. **e**, Pathway enrichment analysis of proteins up or down-regulated in vertical versus radial growth melanoma cells. Enrichment results were obtained with the ClusterProfiler R package^36^ based on an FDR < 0.05.

Decoding the molecular alterations in melanoma development and progression is key to identifying therapeutic vulnerabilities in this highly metastatic disease. With pathogenic mutations in melanoma largely catalogued^27–29^, we set out to directly study spatially resolved proteomes of distinct cellular phenotypes of melanoma progression (Fig. 5a,b and Extended Data Fig. 5a,b). We co-stained FFPE-embedded primary tumor material preserved for 17 years with two markers, SOX10 and CD146, to map melanoma cells. As overexpression of CD146 is implicated in melanoma progression, and immunotherapy against CD146 targets metastasis^30^, we used CD146 as a disease progression marker in our analysis. ML predicted five classes with clearly defined spatial distribution: class 1, melanoma in situ; class 2, predominantly tumor; class 3, cells of the tumor microenvironment; class 4, enriched in CD146-high regions; and class 5, enriched in CD146-low regions. We used high-content imaging to determine the required number of cells to identify statistically and analytically robust cellular phenotypes for precise cell type and state isolation within a spatial region. For this reason, we typically collected around 100 cells per sample (Methods). Including replicates, we isolated and profiled 27 different samples obtained from seven unique regions of the same tissue section, including normal melanocytes, melanoma in situ and primary melanoma from the radial and vertical growth phases (Fig. 5a–d). We found high quantitative reproducibility among biological replicates, resulting in disease state and region-specific proteomes (Fig. 5e–g). Pre-cancerous (melanoma in situ) and primary melanoma showed differences in proteins involved in immune cell signaling and cell metabolism and coincided with reduced melanogenesis (Supplementary Table 7 and Extended Data Fig. 5d). The advanced stages (radial and vertical melanoma growth phase) showed well-defined activation of metabolic activation along with disease progression, a known hallmark of human cancers^31^. Expression of proteins involved in oxidative phosphorylation and mitochondria function gradually increased from melanocytes, melanoma in situ to invasive melanoma, indicating a dependency on mitochondrial respiration in the advanced tumor stages (Fig. 5h–j, Extended Data Fig. 5c and Supplementary Tables 7–9). Conversely, proteins involved in antigen presentation and interferon response were downregulated when compared to melanoma in situ (Fig. 5h–j and Supplementary Tables 7–9), in line with immune evasion strategies in melanoma^32^.

Melanoma progression is a stepwise process involving radial and vertical growth phases. The direct comparison of these spatially defined regions of the same phenotype (class 4 cells) further highlighted critical features of cancer metastasis, such as extracellular matrix (ECM) remodeling (for example, collagen degradation) and upregulated PDGF signaling^33^ (Fig. 5k,l, Extended Data Fig. 5e and Supplementary Table 10). These tumor-driven changes support growth, increase migration of tumor cells and remodel the ECM to facilitate metastasis to distant organs via adjacent blood vessels^33^. DVP also discovered a significant upregulation of mRNA splicing in the vertical compared to the radial growth phase. Pro-oncogenic alternative splicing has recently become a therapeutic strategy in oncology^34^, and these tumors often present immunogenic neoantigens^35^. The increase in splicing coincided with a significant downregulation of immune-related signaling (interferon signaling and antigen presentation) (Fig. 5l and Supplementary Table 10), suggesting the transition from an immunogenic ‘hot’ to a ‘cold’ tumor zone in the vertical growth phase within the same tumor section. Clearly, DVP spatially resolved tumor heterogeneity by localizing tumor-related mRNA splicing, immune responses and ECM remodeling pathways in different regions.

## Discussion

DVP combines imaging technologies with unbiased proteomics to quantify the number of expressed proteins in a given cell, map tissue or cell-type-specific proteomes or to identify targets for future drugs and diagnostics. We showed how our analyses describe a rich ‘microcosm in a slide’, uncovering key pathways dysregulated in cancer progression and effectively extending ‘digital pathology’ by a molecular dimension. It is broadly applicable to any biological system that can be microscopically imaged, from cell culture to pathology. As a single slide can encompass hundreds of thousands of cells, DVP can discover and characterize rare cell states and interactions. In contrast to single-cell transcriptomics, DVP can readily analyze the ECMʼs subcellular structures and spatial dynamics. With further improvements in proteomics technology, DVP should also be suited to study proteoforms and post-translational modifications at a single-cell-type level.

## Methods

### Patient samples and ethics

We collected archival FFPE tissue samples of salivary gland acinic cell carcinoma and melanoma from the Department of Pathology, Zealand University Hospital, in Roskilde, Denmark. Melanoma tissue was from a 51-year-old male and located at the left upper chest. TNM stage at diagnosis was T3aN1M0. The histological subtype was superficial spreading melanoma; the Clark level was 4; and the Breslow thickness was 2.27 mm. Tumor immune infiltration was categorized as non-brisk. The FFPE sample was 17 years old. The patient experienced recurrence at different locations 17 months after diagnosis and died after 71 months. The acinic cell carcinoma was removed from the right parotid gland of a 29-year-old male. There was no sign of mitosis, necrosis de-differentiation or perineural or intravascular growth. The tumor cells were positive in EpCAM, CK7, DOG1 and SOX10. Mammaglobin was negative. The sample was 4 years old, and the patient is currently disease-free. The study was carried out in accordance with institutional guidelines under approval by the local Medical Ethics Review Committee (SJ-742) and the Data Protection Agency (REG-066-2019) and in agreement with Danish law (Medical Research Involving Human Subjects Act). The fallopian tube tissue shown in Fig. 2 is from a 64-year-old female and was macroscopically and histologically normal appearing. All patients consented before surgery. Patient-derived tissues were obtained fresh or paraffin-embedded according to an approved institutional review board protocol (13372B) at the University of Chicago hospital. In accordance with the Medical Ethics Review Committee approval, all FFPE human patient tissue samples were exempted from consent, as these studies used existing archived pathological specimens. Human tissue specimens were assessed by a board-certified pathologist.

### Cell lines

The human osteosarcoma cell line U2OS was grown in DMEM (high glucose, GlutaMAX) containing 10% FBS and penicillin–streptomycin (Thermo Fisher Scientific).

The U2OS FUCCI cells were kindly provided by Atsushi Miyawaki^14^. These cells are endogenously tagged with two fluorescent proteins fused to the cell cycle regulators CDT1 (mKO2-hCdt1^+^) and geminin (mAG-hGem^+^). CDT1 accumulates during the G1 phase, whereas geminin accumulates in the S and G2 phases, allowing cell cycle monitoring. The cells were cultivated at 37 °C in a 5.0% CO_2_ humidified environment in McCoy’s 5A (modified) medium GlutaMAX supplement (Thermo Fisher Scientific, 36600021) supplemented with 10% FBS (VWR) without antibiotics.

U2OS cells stably expressing a membrane-targeted form of eGFP were generated by transfection with plasmid Lck-GFP (Addgene, 61099 (ref. ^37^)) and culturing in selection medium (DMEM medium containing 10% FBS, penicillin–streptomycin and 400 μg ml^−1^ of Geneticin) under conditions of limited dilution to yield single colonies. A clonal cell line with homogenous and moderate expression levels of Lck-eGFP at the plasma membrane was established from a single colony.

All cell lines were tested for mycoplasma (MycoAlert, Lonza) and authenticated by STR profiling (IdentiCell).

### IHC staining on membrane slides

Membrane PEN slides 1.0 (Zeiss, 415190-9041-000) were treated with UV light for 1 hour and coated with APES (3-aminopropyltriethoxysilane) using VECTABOND reagent (Vector Labs, SP-1800-7) according to the manufacturer’s protocol. FFPE tissue sections were cut (2.5 µm), air dried at 37 °C overnight and heated at 60 °C for 20 minutes to facilitate better tissue adhesion. Next, sections were deparaffinized, rehydratrated and loaded wet on the fully automated instrument Omnis (Dako). Antigen retrieval was conducted using Target Retrieval Solution pH 9 (Dako, S2367) diluted 1:10 and heated for 60 minutes at 90 °C. Single stain for EpCAM (Nordic BioSite, clone BS14, BSH-7402-1, dilution 1:400) and sequential double stain for SOX10/CD146 (SOX10, Nordic BioSite, clone BS7, BSH-7959-1, dilution 1:200; CD146, Cell Marque, clone EP54, AC-0052, dilution 1:400) was performed, and slides were incubated for 30 minutes (32 °C). After washing and blocking of endogenous peroxidase activity, the reactions were detected and visualized using EnVision FLEX, High pH kit (Dako, GV800 and GV809/GV821) according to the manufacturer’s instructions. In the double stain, EnVision DAB (Dako, GV825) and EnVision Magenta (Dako, GV900) substrate chromogen systems were used for visualization of CD146 and SOX10, respectively. Finally, slides were rinsed in water, counterstained with Mayerʼs hematoxylin and air dried without mounting.

### IHC staining for validation of DVP studies

FFPE tissue sections were cut (2.5 µm), placed on coated slides (Agilent/Dako, K8020) and air dried vertically before heating at 60 °C for 20 minutes to facilitate tissue adhesion. Next, slides were loaded on the fully automated instrument Omnis. Sections were dewaxed, and antigen retrieval was conducted using Target Retrieval Solution High pH (Agilent/Dako, GV804, diluted 1:50) at 97 °C for 24 minutes. Subsequently, the sections were incubated with the primary antibodies. We selected antibodies assessed and approved by a board-certified consultant pathologist. Proto-oncogene tyrosine protein kinase SRC/c-Src (Cell Signaling Technology, clone 36D10, 2109, dilution 1:3,200), fatty acid synthase/FASN (Cell Signaling Technology, clone C20G5, 3180, dilution 1:100), calponin-1/CNN1 (Cell Marque, clone EP63, AC-0060, dilution 1:300) and cytokeratin 5/CK5 (Leica Biosystems, clone XM26, NCL-L-CK5, dilution 1:200) for 30 minutes at 32 °C. After washing and blocking of endogenous peroxidase activity, the reactions were detected and visualized using EnVision FLEX, High pH kit (Agilent/Dako, GV800 and GV809/GV821) according to the manufacturer’s instructions. Finally, slides were rinsed in water, counterstained with Mayerʼs hematoxylin and cover-slipped.

### Immunofluorescence staining

Cells were first incubated with 5-ethynyl-2′-deoxyuridine (EdU) for 20 minutes and then fixed for 5 minutes at room temperature with 4% paraformaldehyde (PFA) and washed three times with PBS. Cells were then permeabilized with PBS/0.2% Triton X-100 for 2 minutes on ice and washed three times with PBS. Cells were then stained with an EdU labeling kit (Life Technologies) and counterstained with Hoechst 33342 for 10 minutes. Slides were mounted with GB mount (GBI Labs, E01-18).

For validation experiments (Extended Data Fig. 3), 96-well glass-bottom plates (Greiner SensoPlate Plus, Greiner Bio-One) were coated with 12.5 µg ml^−1^ of human fibronectin (Sigma-Aldrich) for 1 hour at room temperature. Immunocytochemistry was carried out following an established protocol^38^. Then, 8,000 U2OS cells were seeded in each well and incubated in a 37 °C and 5% CO_2_ environment for 24 hours. Cells were washed with PBS, fixed with 40 µl of 4% ice-cold PFA and permeabilized with 40 µl of 0.1 Triton X-100 in PBS for 3×5 minutes. Rabbit polyclonal HPA antibodies targeting the proteins of interest were diluted in blocking buffer (PBS + 4% FBS) at 2–4 µg ml^−1^ along with primary marker antibodies (see below) and incubated overnight at 4 °C. Cells were washed with PBS for 4×10 minutes and incubated with secondary antibodies (goat anti-rabbit Alexa Fluor 488 (A11034, Thermo Fisher Scientific), goat anti-mouse Alexa Fluor 555 (A21424, Thermo Fisher Scientific) and goat anti-chicken Alexa Fluor 647 (A21449, Thermo Fisher Scientific)) in blocking buffer at 1.25 µg ml^−1^ for 90 minutes at room temperature. Cells were counterstained in 0.05 µg ml^−1^ of DAPI for 15 minutes, washed with for 4×10 minutes and mounted in PBS.

Primary antibodies used were as follows:

For C7orf50 cell cycle validation: mouse anti-ANLN at 1.25 µg ml^−1^ (amab90662, Atlas Antibodies)

Mouse anti CCNB1 at 1 µg ml^−1^ (610220, BD Biosciences)

Rabbit anti-C7orf50 at 1 µg ml^−1^ (HPA052281, Atlas Antibodies)

For human fallopian tube tissue, FFPE tissue sections (2.5 µm) were mounted and pre-processed as described above. Thereafter, tissue was dewaxed by washing 2×2 minutes in 100% xylene, followed by a series of 100%, 95% and 70% ethanol for 1 minute, respectively, and 3×1 minute in ddH_2_O. Antigen retrieval was performed in a water bath employing EDTA retrieval buffer (1 mM EDTA, 0.05% Tween 20, pH 8.0) at 95 °C for 1 hour. Subsequent to a cooling phase of 1 hour at room temperature, blocking was conducted with 10% goat serum in TBST for 1 hour at room temperature. Primary antibodies targeting FOXJ1 (mouse, dilution 1:200, 14-9965-80, Invitrogen) and EpCAM (rabbit, dilution 1:200, 14452, Cell Signaling Technology) were diluted in 10% goat serum and incubated overnight at 4 °C in a humidified chamber. Tissue specimens were washed 5× in TBST and secondary antibodies for the visualization of FOXJ1 (Alexa Fluor 647 goat anti-mouse, dilution 1:200, A21235, Invitrogen) and EpCAM (Alexa Fluor 555 goat anti-rabbit, dilution 1:200, A21428, Invitrogen), and SYTO 10 for nuclear visualization (10624243, Invitrogen) was applied for 1 hour at room temperature in darkness. Samples were washed 5× in TBST, followed by 2× in TBS and cover-slipped for high-content imaging.

### High-resolution microscopy

Images of immunofluorescence-labeled cell cultures were acquired using an AxioImager Z.2 microscope (Zeiss), equipped with wide-field optics, a ×20, 0.8 NA dry objective and a quadruple-band filter set for Hoechst, FITC, Cy3 and Cy5 fluorescent dyes. Wide-field acquisition was performed using the Colibri 7 LED light source and an AxioCam 702 mono camera with 5.86 μm per pixel. Z-stacks with 19 z-slices were acquired at 3-mm increments to capture the optimal focus plane. Images were obtained automatically with Zeiss ZEN 2.6 (blue edition) at non-saturating conditions (12-bit dynamic range).

IHC images from salivary gland and melanoma tissue were obtained using the automated slide scanner Zeiss Axio Scan.Z1 for bright-field microscopy. Bright-field acquisition was obtained using the VIS LED light source and a CCD Hitachi HV-F202CLS camera. PEN slides were scanned with a ×20, 0.8 NA dry objective yielding a resolution of 0.22 mm per pixel. Z-stacks with eight z-slices were acquired at 2-mm increments to capture the optimal focus plane. Color images were obtained automatically with Zeiss ZEN 2.6 (blue edition) at non-saturating conditions (12-bit dynamic range).

#### Wide-field fluorescence microscopy for validation of cell-cycle-dependent C7orf50 expression

Cells were imaged on a Leica Dmi8 wide-field microscope equipped with a 0.8 NA, ×40 air objective and a Hamamatsu Flash 4.0 V3 camera using LAS X software. The segmentation of each cell was performed using Cell Profiler software^8^ using DAPI for nuclei segmentation. The mean intensity of the target protein and the cell cycle marker protein was measured in the nucleus. The cells were grouped into the G1 and G2 phases of the cell cycle by using the 0.2 and 0.8 quantile of ANLN or CCNB1 intensity levels in the nucleus, and cell-cycle-dependent expression of C7orf50 was validated by comparing differences in expression levels between G1 and G2 cells.

### LMD

To excise cells or nuclei, we used the Leica LMD7 system, which we adapted for automated single-cell automation. High cutting precision was achieved using an HC PL FLUOTAR L ×63/0.70 (tissue) or ×40/0.60 (cell cultures) CORR XT objective. We used the Leica Laser Microdissection V 8.2.3.7603 software (adapted for this project) for full automated excision and collection of contours. For FFPE tissue proteome analysis, we collected 50–100 cells per sample (total area collected × slide thickness / average mammalian cell volume of 2,000 µm^3^; BNID 100434), in agreement with estimations in spatial transcriptomics analysis^39^.

Leica LMD7 cutting accuracy (Leica R&D, patent EP1276586)

For ×150 objective: $${\frac{10}{150}} = 0.07\,\upmu{\mathrm{m}}$$

### Segmentation methods and accuracy evaluation

nucleAIzer^3^ models were integrated into BIAS and customized for these experiments by retraining and refining the nucleus and cytoplasm segmentation models. First, style transfer^5^ learning was performed as follows. Given a new experimental scenario such as our melanoma or salivary gland tissue sections stained immunohistochemically, the acquisition of which produces such an image type that no annotated training data exist for, preventing efficient segmentation with even powerful DL methods. With an initial segmentation or manual contouring by experts (referred to as annotation), a small mask dataset is acquired (masks represent, for example, nuclei), which is used to generate new (synthetic) mask images such that the spatial distribution, density and morphological properties of the generated objects (for example, nuclei) are similar to those measured on the annotated images. The initial masks and their corresponding microscopy images are used to train an image style transfer model that learns how to generate the texture of the microscopy images on the masks, marking objects using GANs^40^ (generative adversarial networks): foreground to mimic, for example, nuclei, and background for surrounding, for example, tissue structures. Parallelly, artificial masks of either nucleus or cytoplasm objects were created and input to the image style transfer learning network that generated realistic-looking synthetic microscopy images with the visual appearance of the original experiment. Hence, with this artificially created training data (synthetic microscopy images and their corresponding, also synthetic, masks), their applied segmentation model, Mask R-CNN, is prepared for the new image type and can accurately segment the target compartments.

We benchmarked the accuracy of the segmentation approach on a fluorescent Lck-U2OS cell line as well as tissue samples of melanoma, salivary gland and fallopian tube and compared results to three additional methods, including two DL approaches—unet4nuclei (denoted as M_1_ in Fig. 2a and S1)^6^ and Cellpose (M_3_)^7^—alongside a widely used, conventional adaptive threshold-based and object splitting-based application (M_2_)^8^. We note that M_1_ is not intended for cytoplasm segmentation (see details in ref. ^6^ and below). Segmentation accuracy according to the F1 metric is displayed as bar plots (Fig. 2b, Extended Data Fig. 1a, Table 1 and Supplementary Table 1), and visual representation in a color-coded manner is also provided.

unet4nuclei^6^ is optimized to segment nuclei on cell culture images; Cellpose^7^ is an approach intended for either nucleus or cytoplasm segmentation on various microscopy image types; and CellProfiler^8^ is a conventional threshold-based and object splitting-based software broadly used in the bioimage analysis community. unet4nuclei, as its name suggests, is primarily intended for nucleus segmentation and uses a U-Net-based network after pre-processing of input images and then post-processes detected objects. Cellpose uses a vector flow representation of instances, and its neural network (also based on U-Net) predicts and combines horizontal and vertical flows. unet4nuclei has successfully been applied in nucleus segmentation of cell cultures, whereas Cellpose is able to generalize well on various image modalities even outside microscopy and can be used to segment nuclei and cytoplasms. However, as most segmentation methods, neither is able to adapt to a new image domain, such as a particular experiment type (for example, IHC salivary gland tissue), without re-training on newly created ground truth annotations. On the contrary, our segmentation algorithm (nucleAIzer^3^) is able to do so via the image style transfer approach mentioned above. Obviously, conventional algorithms cannot adapt either; thus, they need to be re-parameterized for each experiment. For the evaluation, an expert CellProfiler user was asked to optimize a pipeline for each sample type to the best possible segmentation result, and then all images per sample type were segmented with one pipeline (corresponding to the given sample).

We evaluated our segmentation performance (and comparisons) according to the F1 score metric calculated at the 0.7-IoU (intersection over union) threshold. IoU, also known as Jaccard index, was calculated from the overlapping region of the predicted (segmented) object with its corresponding ground truth (real) object at a given threshold (see formulation below). True-positive (TP), false-positive (FP) and false-negative (FN) objects were counted accordingly, if they had an IoU greater than the threshold *t* (in our case, 0.7), to yield the F1 score at this threshold (see formulation below). Segmentation evaluation was performed on 10–20 randomly selected images sampled from visually distinct regions for each sample type (U2OS cells and melanoma, salivary gland and fallopian tube tissues) to show robustness, compared to ground truth annotations drawn by experts using AnnotatorJ^41^. We included images from all relevant regions of each sample—for example, duct cells, acini cells, cells without any membrane staining and lymphocytes—in the salivary gland tissue, and similarly for the other samples as well, to ensure robustness. Outlines or contours of all visible objects (nucleus or cytoplasm) were drawn individually and then exported to mask images in the same format that the segmentation yielded (instance segmentation masks with increasing gray intensities by objects). The ground truth masks were solely used in evaluation; the aforementioned image style transfer learning was trained on automatically fetched masks of the new experiments. Considering the mean F1 scores measured, we conclude that the applied DL-based segmentation method^3^ available in BIAS produced segmentations on both nucleus and cytoplasm level in a higher quality than the compared methods (see results in Fig. 2a,b and Extended Data Fig. 1a).

$$Jaccard\,index = \frac{{\left| {x \cap y} \right|}}{{\left| {x \cup y} \right|}} = \frac{{\left| {x \cap y} \right|}}{{\left| x \right| + \left| y \right| - \left| {x \cap y} \right|}}$$

$$precision(t) = \frac{{TP(t)}}{{TP(t) + FP(t)}}$$

$$recall(t) = \frac{{TP(t)}}{{TP(t) + FN(t)}}$$

$$F1\,score(t) = 2 \cdot \frac{{precision(t) \cdot recall(t)}}{{precision(t) + recall(t)}}$$

Our evaluation results of nucleus and cell body segmentation on melanoma, salivary gland and fallopian tube epithelium tissues and U2OS cells is presented in Table 1.

These results correlate with our pevious study^3^ that showed superior performance of nucleAIzer on various microscopy image data modalities (fluorescent cell culture, hematoxylin and eosin tissue and further experimental scenarios) compared to multiple segmentation approaches, including, for example, M_2_ and ilastik^9^.

We also note that previous methods, such as CellProfiler or ilastik, can perform accurate segmentation of cells; moreover, the performance of M_2_ on tissue nucleus segmentation is remarkable. On the other hand, robust methods (for example, DL-based) offer the convenience of not needing to reset most parameters when working on images from a different sample or type.

### Sample preparation for MS

Cell culture (nuclei or whole cells) and tissue samples were collected by automated LMD into 384-well plates (Eppendorf, 0030129547). For the collection of different U2OS nuclei classes (Fig. 3 and Extended Data Figs. 2 and 3), we normalized nuclear size differences (resulting in different total protein amounts) by the number of collected objects per class. On average, we collected 267 nuclei per sample. For FFPE tissue samples of salivary gland and melanoma (2.5-µm-thick sections cut with a microtome), an area of 80,000–160,000 µm^2^ per sample was collected for an estimated number of 100–200 cells based on the average HeLa cell volume of 2,000 μm^3^ (BNID 100434).

Next, 20 µl of ammonium bicarbonate (ABC) was added to each sample well, and the plate was closed with sealing tape (Corning, CLS6569-100EA). After vortexing for 10 seconds, plates were centrifuged for 10 minutes at 2,000*g* and heated at 95 °C for 30 minutes (cell culture) or 60 minutes (tissue) in a thermal cycler (Bio-Rad S1000 with 384-well reaction module) at a constant lid temperature of 110 °C. Then, 5 µl of 5× digestion buffer (60% acetonitrile in 100 mM ABC) was added, and samples were heated at 75 °C for another 30 minutes. Samples were shortly cooled down, and 1 µl of LysC was added (pre-diluted in ultra-pure water to 4 ng µl^−1^) and digested for 4 hours at 37 °C in the thermal cycler. Subsequently, 1.5 µl of trypsin was added (pre-diluted in ultra-pure water to 4 ng µl^−1^) and incubated overnight at 37 °C in the thermal cycler. The next day, digestion was stopped by adding trifluoroacetic acid (TFA, final concentration 1% v/v), and samples were vacuum dried (approximately 1.5 hours at 60 °C). Then, 4 µl of MS loading buffer (3% acetonitrile in 0.2% TFA) was added, and the plate was vortexed for 10 seconds and centrifuged for 5 minutes at 2,000*g*. Samples were stored at −20 °C until liquid chromatography–mass spectrometry (LC–MS) analysis.

### High-pH reversed-phase fractionation

We used high-pH reversed-phase fractionation to generate a deep U2OS cell precursor library for data-independent MS analysis (below). Peptides were fractionated at pH 10 with the spider-fractionator^42^. Next, 30 μg of purified peptides was separated on a 30-cm C18 column in 100 minutes and concatenated into 12 fractions with 90-second exit valve switches. Peptide fractions were vacuum dried and reconstituted in MS loading buffer for LC–MS analysis.

### LC–MS analysis

LC–MS analysis was performed with an EASY-nLC-1200 system (Thermo Fisher Scientific) connected to a modified trapped ion mobility spectrometry quadrupole time-of-flight mass spectrometer with about five-fold-higher ion current (timsTOF Pro, Bruker Daltonik) with a nano-electrospray ion source (CaptiveSpray, Bruker Daltonik). The autosampler was configured for sample pick-up from 384-well plates.

Peptides were loaded on a 50-cm in-house-packed HPLC column (75-µm inner diameter packed with 1.9-µm ReproSil-Pur C18-AQ silica beads, Dr. Maisch).

Peptides were separated using a linear gradient from 5–30% buffer B (0.1% formic acid and 80% ACN in LC–MS-grade water) in 55 minutes, followed by an increase to 60% for 5 minutes and a 10-minute wash in 95% buffer B at 300 nl min^−1^. Buffer A consisted of 0.1% formic acid in LC–MS-grade water. The total gradient length was 70 minutes. We used an in-house-made column oven to keep the column temperature constant at 60 °C.

Mass spectrometric analysis was performed as described in Brunner et al., either in data-dependent (ddaPASEF) (Fig. 4) or data-independent (diaPASEF) mode (Figs. 2, 3 and 5). For ddaPASEF, one MS1 survey TIMS-MS and ten PASEF MS/MS scans were acquired per acquisition cycle. Ion accumulation and ramp time in the dual TIMS analyzer was set to 100 ms each, and we analyzed the ion mobility range from 1/K_0_ = 1.6 Vs cm^−^^2^ to 0.6 Vs cm^−^^2^. Precursor ions for MS/MS analysis were isolated with a 2-Th window for *m*/*z* < 700 and 3-Th for *m*/*z* > 700 in a total *m*/*z* range of 100–1.700 by synchronizing quadrupole switching events with the precursor elution profile from the TIMS device. The collision energy was lowered linearly as a function of increasing mobility starting from 59 eV at 1/K_0_ = 1.6 Vs cm^−^^2^ to 20 eV at 1/K_0_ = 0.6 Vs cm^−^^2^. Singly charged precursor ions were excluded with a polygon filter (otof control, Bruker Daltonik). Precursors for MS/MS were picked at an intensity threshold of 1.000 arbitrary units (a.u.) and re-sequenced until reaching a ‘target value’ of 20.000 a.u., taking into account a dynamic exclusion of 40-second elution. For data-independent analysis, we made use of the correlation of ion mobility with *m*/*z* and synchronized the elution of precursors from each ion mobility scan with the quadrupole isolation window. The collision energy was ramped linearly as a function of the ion mobility from 59 eV at 1/K_0_ = 1.6 Vs cm^−^^2^ to 20 eV at 1/K_0_ = 0.6 Vs cm^−2^. We used the ddaPASEF method for library generation.

### Data analysis of proteomic raw files

Mass spectrometric raw files acquired in ddaPASEF mode (Fig. 4) were analyzed with MaxQuant (version 1.6.7.0)^43,44^. The UniProt database (2019 release, UP000005640_9606) was searched with a peptide spectral match and protein-level FDR of 1%. A minimum of seven amino acids was required, including N-terminal acetylation and methionine oxidation as variable modifications. Due to omitted reduction and alkylation, cysteine carbamidomethylation was removed from fixed modifications. Enzyme specificity was set to trypsin with a maximum of two allowed missed cleavages. First and main search mass tolerance was set to 70 p.p.m. and 20 p.p.m., respectively. Peptide identifications by MS/MS were transferred by matching four-dimensional isotope patterns between the runs (MBR) with a 0.7-minute retention time match window and a 0.05 1/K_0_ ion mobility window. Label-free quantification was performed with the MaxLFQ algorithm^45^ and a minimum ratio count of 1.

For diaPASEF measurements (Figs. 2, 3 and 5), raw files were analyzed with DIA-NN^46^ (version 1.8). To generate a project-specific spectral library, a 24-fraction high-pH reversed-phase fractionated precursor library was created from the same tissue specimen and acquired in ddaPASEF mode, as described above. Raw files were analyzed with MSFragger^47^ under default settings (with the exception that cysteine carbamidomethylation was removed from fixed modifications) to generate the library file used in DIA-NN. The library consisted of 90,056 precursors, 79,802 elution groups and 7,765 protein groups.

### Bioinformatic analysis

Proteomics data analysis was performed with Perseus^48^ and within the R environment (﻿https://www.r-project.org/). MaxQuant output tables were filtered for ‘Reverse’, ‘Only identified by site modification’ and ‘Potential contaminants’ before data analysis. Data were stringently filtered to keep proteins with only 30% or less missing values (those displayed as 0 in MaxQuant output). Missing values were imputed based on a normal distribution (width = 0.3; downshift = 1.8) before statistical testing. PCA was performed in R. For multi-sample (ANOVA) or pairwise proteomic comparisons (two-sided unpaired *t*-test), we applied a permutation-based FDR of 5% to correct for multiple hypothesis testing. An *s*_*0*_ value^49^ of 0.1 was used for the pairwise proteomic comparison in Figs. 2h and 4e. Pathway enrichment analysis was performed in Perseus (Supplementary Tables 2, 3, 5 and 9; Fisher’s exact test with Benjamini–Hochberg FDR of 0.05) or ClusterProfiler^36^ (Supplementary Tables 7 and 10), the ReactomePA package^50^ and the WebGestalt gene set analysis toolkit (WebGestaltR)^51^, with an FDR filter of 0.05, respectively. Minimum category size was set to 20 and maximum size to 500.

### Microscopy and proteomics data integration

To visualize combined microscopy and MS-based proteomics results, we exported the spatial data files for each predicted class from the BIAS software. This export generates .xml output files with the geometry and location of cells within a class. We used Python to extract this information and aggregated it into a data frame. We then plotted the centroid (*x–y* coordinates) of each cell in a scatterplot and overlapped proteomics data. To visualize protein functional results in spatial context, we performed a REACTOME pathway enrichment analysis on the generated proteomics results and used normalized enrichment scores (z-scores) as a color gradient reflecting overrepresentation of a given pathway.

### Reporting Summary

Further information on research design is available in the Nature Research Reporting Summary linked to this article.

## Online content

Any methods, additional references, Nature Research reporting summaries, source data, extended data, supplementary information, acknowledgements, peer review information; details of author contributions and competing interests; and statements of data and code availability are available at 10.1038/s41587-022-01302-5.

## Supplementary information

Supplementary Information BIAS information

Reporting Summary

Supplementary Video 1 Automated LMD single-nucleus isolation

Supplementary Video 2 Automated LMD single-cell isolation

Supplementary Table 1 Benchmark accuracy of segmentation approach using the F1 metric and comparison of results to three additional methods

Supplementary Table 2 Pathway enrichment analysis for proteins significantly higher in ciliated cells compared to secretory fallopian tube epithelial cells based on a Fisher’s exact test with a Benjamini–Hochberg FDR < 0.05

Supplementary Table 3 Pathway enrichment analysis results for proteins differentially regulated across nucleus classes based on a Fisher’s exact test with a Benjamini–Hochberg FDR < 0.05

Supplementary Table 4 Proteins differentially regulated across nucleus classes based on ANOVA analysis with a permutation-based FDR < 0.05

Supplementary Table 5 Pathway enrichment analysis results for proteins differentially regulated between acinar and duct cells of the healthy salivary gland based on a two-sided (two-sample) Wilcoxon–Mann–Whitney test with a Benjamini–Hochberg FDR < 0.05

Supplementary Table 6 Proteins differentially regulated between acinic cell carcinoma and normal acinar cells. A two-sided *t*-test was performed with a permutation-based FDR < 0.05

Supplementary Table 7 Gene set enrichment analysis of Reactome Pathways (ReactomePA) for melanoma in situ versus primary melanoma. Pathways with an FDR-adjusted *P* value of <0.05 are shown

Supplementary Table 8 Proteins differentially regulated among all cell classes of the primary melanoma based on ANOVA analysis with a permutation-based FDR < 0.05

Supplementary Table 9 Pathway enrichment analysis results for proteins in the two highlighted heat map clusters of Fig. 5i based on a Fisher’s exact test with a Benjamini–Hochberg FDR < 0.05

Supplementary Table 10 Gene set enrichment analysis of Reactome Pathways (ReactomePA) for melanoma cells of the vertical versus radial growth phase. Pathways with an FDR-adjusted *P* value of <0.05 are shown

Supplementary Table 11 Links to open-source repositories

## Data Availability

The mass spectrometry proteomics data have been deposited to the ProteomeXchange Consortium via the PRIDE partner repository^52^ with the dataset identifier PXD023904. BIAS raw data, image raw data, a demo dataset and online material of how to install BIAS and reproduce our work can be accessed at the European Bioinformatics Institute BioStudies database^53^ (https://www.ebi.ac.uk/biostudies/) with the accession number S-BSST820. We used the UniProt database (2019 release, UP000005640_9606, https://www.uniprot.org) for all mass spectrometric raw file searches.

## Code availability

A free compiled version of BIAS with limited high-throughput capabilities is available at the BioStudies Archive (accession number S-BSST820), containing all features applied in the described workflows. Several major components of our work are available in open-source repositories (Supplementary Table 11).
