# Supplementary material for: Deep Visual Proteomics defines single-cell identity and heterogeneity
Source: Nat Biotechnol. 2022 May 19;40(8):1231–40. doi: 10.1038/s41587-022-01302-5 (PMC9371970; doi:10.1038/s41587-022-01302-5)
Supplement: Supplementary file 1 — BIAS information [file 41587_2022_1302_MOESM1_ESM.pdf]

nature portfolio

https://doi.org/10.1038/s41587-022-01302-5

## Supplementary information

# Deep Visual Proteomics defines single-cell identity and heterogeneity

In the format provided by the
authors and unedited

SUPPLEMENTARY INFORMATION

## Biological Image Analysis Software (BIAS) introduction

A typical image analysis workflow in BIAS consists of multiple images processing steps, including image preprocessing, object segmentation, contour post-processing, feature extraction and statistical analysis, supervised or unsupervised machine learning methods for phenotype classification, cell selection and cell extraction (by a selected or supported micro-dissection microscope). For each workflow step BIAS provides conveniently customizable modules.

Images were captured with a Zeiss Axio Scan.Z1 or AxioImager Z.2 microscope, both are supported by the analysis software with preservation of correct spatial information (spatial topology and size). Other types of microscopes with similar support include 3DHistech, Hamamatsu, GE IN Cell, Molecular Devices ImageXpress Micro, Leica SP, Perkin Elmer Opera and Operetta. It is also possible to import standard image files with editable image orientation and resolution. Illumination-correction algorithm, primarily CIDRE[36] (within BIAS) were applied where it was necessary to solve the frequent 'vignetting' effect observable in raw microscopy images.

Image preprocessing was followed by deep learning-based nucleus and cell segmentation modules (see segmentation methods and accuracy evaluation) further refined by unary and binary morphological operators (e.g.: dilation, erosion, cavity filling, addition and subtraction). For example, subtraction can be used to calculate the cytoplasm-only region from cell and nuclei masks.

Results of different segmentation algorithms may be connected by a linking module to form complex structures, e.g. an abstract cell object might be constructed from a segmented nucleus, cytoplasm and proteins where each component can be analyzed individually or as a whole. Objects were forwarded to the feature extraction modules, configurable to extract properties from the selected image channels and cell components. A multitude of features can be retrieved from the image and contour data, such as shape (e.g.: area, perimeter, form factor, solidity etc.), intensity (e.g.: min, max, mean, total) or texture (e.g.: Haralick features) and represented in a feature matrix[37]. Features to be extracted may vary by experiments according to their specific requirements each, potentially containing up to hundreds or a few thousands per cell depending on the configuration. Features from the neighboring regions of each cell can be incorporated as well, to further improve accuracy where local neighborhoods might also contain valuable information for the cell phenotypes (such as in tissues)[38]. The resulting feature matrices can be analyzed internally or exported to a 3rd party tool[39]. Subsequently reimport of extended feature matrices into BIAS is also possible to extend the statistical capabilities or simply to visualize the data in the plate overview.

Internal analysis tools include simple, value-based statistics, manual gating, automatic feature space clustering and interactive supervised machine learning; additionally, these may also be combined. Final cell selection can depend on simple, value-based statistics or complex queries searching in multiple feature and classification matrices.

With manual gating, two features can be represented in a two-dimensional coordinate system and with cluster centers defined manually, samples are displayed at their actual position in the coordinate system. K-Means clustering can automatically find a fixed number of cluster centers in the feature space with an arbitrary number of dimensions.

During supervised machine learning, the biologist defines the phenotypes of interest and provides training samples (usually around a hundred samples for each class). Training is iterative and interactive, refinable and new phenotypes may be identified using an active learning technique[10]. A cross validation tool is provided to continuously monitor accuracy, so that when a satisfactory threshold is reached, all other cells in the whole experiment are classified. Different machine learning approaches are implemented in the BIAS software for various experimental needs. Such methods are 1) Multilayer perceptron (a feedforward artificial neural network), 2) Support-vector machine (separates the feature space by hyperplanes between the training samples), 3) Random forest (a number of decision trees trained to separate the training data into classes) and 4) Logistic regression (a statistical model that determines the probability of passing or failing the criteria of a certain class). These classification algorithms can analyze and classify tens of thousands of cells in a matter of seconds.

Results of the feature extraction and classification phases can be summarized in the statistics module, in addition it also provides an interface where custom queries can be written in SQL language and executed on the cell information database containing feature and classification data for all cells in the experiment. Cross queries between different classification and feature matrices are also supported. Query templates and wizards are provided for the most common questions.

The queries might be as simple as e.g. listing N items that have the highest value in a selected feature, or rather complex e.g. to calculate the sum and ratio of the areas of cells belonging to different classes). Results can be represented in graphs, heatmaps or used to filter cells for capturing by a suitable microscope.

The visualization tool supports a virtually unlimited number of channels with adjustable intensity window, gamma and look-up-table settings. An interactive, zoomable overview of the whole experiment (let it be a slide or a plate) can be displayed, reflecting the changes in visualization or data processing real-time. The results of all processing steps could be overlaid on it, such as segmentation masks, feature heatmaps or phenotype classification as color-coded segments, etc. A schematic display of the multi-well plate helps the navigation, enabling manual selection of cross-field areas for isolation.

The isolation and collection module uses a registration algorithm based on a marker or point-of-interest (POI) to connect the coordinate systems of the source and the isolation microscopes as well as to transfer the contour points to that of the isolation microscope. The tool supports sorting cells into different collectors and also cutting components in order (e.g. cutting nuclei into a microplate well or collection cap first then remaining cytoplasm into another). Cells of interest can be selected manually or using the statistics module. To preserve object integrity, it allows the user to define cutting offsets or exclude touching regions thereby preventing undesirable laser-induced damage.

## Generic BIAS workflow

A usual, generic BIAS workflow, details may be different between experiments as each experiment comes with their own unique requirements and thus the order and number of modules and parameters. Please consult the uploaded project files for each dataset for the actual parameters to reproduce the results of this publication.

Steps used in the experiments of the paper:

Preprocessing:

- Focus (optional) - to select the sharpest image in the z stack
- Image selection (optional) - to select relevant images on the slide, remove fields that only contain background data

Segmentation:

- Segmentation - Nuclei segmentation with deep learning. The actual segmentation model and parameters depend on the experiment (eg. Fluorescent or histological, magnification, average nucleus size, etc)
- Segmentation (optional) - Whole cell segmentation with deep learning.

Segmentation postprocessing:

- Mask operators - dilation (optional) - If cell segmentation is not available, dilation can be applied around the nuclei masks to simulate the cytoplasm area
- Mask operators - relabel (optional) - If the source of the nuclei and the cell masks are independent, relabel can change the label on the secondary (cell) mask to reflect the labels of the primary (nuclei) mask
- Mask operators - complement - Nuclei masks can be removed from the cell masks (simulated or real) to calculate a cytoplasm only mask
- Mask operators - filtering (optional) - Remove masks that are too small or too large or are on the edge of the field. Can prevent double segmentation on scans with overlaps between fields

Analysis preprocessing:

- List creator - Link the cell components (eg. nucleus, cytoplasm, etc) into a single abstract cell object

Analysis:

- Feature extraction - Extract features for all components on all channels. Actual settings depend on the needs of the experiment.
- Neighborhood feature extraction (optional) - Can extract features also from other cells in the cell neighborhood

Phenotype classification:

- Clustering (optional) - Manual gating and unsupervised machine learning. Can detect phenotypes by classifying cells into manually or automatically calculated clusters.
- Machine learning classification (optional) - Supervised, interactive machine learning tool for cell classification.

Finalizing:

- Regions (optional) - Can manually define larger regions on the slide to make further cell selection and extraction easier
- Statistics - Can calculate statistics (based on features or classification results) and recommend cells (also based on features or classification results) for further processing, like single-cell extraction and OMICS
- Contour export - Selects the cells from either the result of a classification or the statistics module for extraction. Generates LMD microscope compatible contour list for laser microdissection.
