# Supplementary material for: Deep Visual Proteomics defines single-cell identity and heterogeneity
Source: Nat Biotechnol. 2022 May 19;40(8):1231–40. doi: 10.1038/s41587-022-01302-5 (PMC9371970; doi:10.1038/s41587-022-01302-5)
Supplement: Supplementary file 2 — Reporting Summary [file 41587_2022_1302_MOESM2_ESM.pdf]

nature research

Corresponding author(s): Andreas Mund, Peter Horvath, and Matthias Mann

Last updated by author(s): Mar 27, 2022

Reporting Summary

Nature Research wishes to improve the reproducibility of the work that we publish. This form provides structure for consistency and transparency in reporting. For further information on Nature Research policies, see our Editorial Policies and the Editorial Policy Checklist.

Statistics

For all statistical analyses, confirm that the following items are present in the figure legend, table legend, main text, or Methods section.

n/a	Confirmed	
☐	☒	The exact sample size (*n*) for each experimental group/condition, given as a discrete number and unit of measurement
☐	☒	A statement on whether measurements were taken from distinct samples or whether the same sample was measured repeatedly
☐	☒	The statistical test(s) used AND whether they are one- or two-sided<br>*Only common tests should be described solely by name; describe more complex techniques in the Methods section.*
☒	☐	A description of all covariates tested
☐	☒	A description of any assumptions or corrections, such as tests of normality and adjustment for multiple comparisons
☐	☒	A full description of the statistical parameters including central tendency (e.g. means) or other basic estimates (e.g. regression coefficient) AND variation (e.g. standard deviation) or associated estimates of uncertainty (e.g. confidence intervals)
☐	☒	For null hypothesis testing, the test statistic (e.g. *F*, *t*, *r*) with confidence intervals, effect sizes, degrees of freedom and *P* value noted<br>*Give P values as exact values whenever suitable.*
☒	☐	For Bayesian analysis, information on the choice of priors and Markov chain Monte Carlo settings
☒	☐	For hierarchical and complex designs, identification of the appropriate level for tests and full reporting of outcomes
☐	☒	Estimates of effect sizes (e.g. Cohen's *d*, Pearson's *r*), indicating how they were calculated

Our web collection on statistics for biologists contains articles on many of the points above.

Software and code

Policy information about availability of computer code

Data collection	Proteomic mass spectrometry data were acquired using Compass Hystar software (Bruker Daltonik GmbH, Germany, version 6.0).<br>AxioImager Z.2 microscope (Zeiss, Germany) for immunofluorescence microscopy. Zeiss Axio Scan.Z1 (Zeiss, Germany) for brightfield microscopy.<br>Segmentation evaluation was performed on 10 or 20 randomly selected images sampled from visually distinct regions for each sample type (U2OS cells, melanoma, fallopian tube, or salivary gland tissues) to show robustness, compared to ground truth annotations drawn by experts using AnnotatorJ (Hollandi, R., et al., Mol. Biol. Cell (2020)). The follwing R packages were used: ReactomePA (version 1.30.0), ggplot2 (version 3.3.1.9000)
Data analysis	Proteomics: MaxQuant (version 1.6.7.0) for dda-PASEF raw files, DIA-NN (version1.8) for dia-PASEF raw files, statistical and bioinformatics analyses were done using Perseus (version 1.6.2.3) or R Studio (version 1.2.5033, R version 3.6.0),<br>Microscopy: ZEN blue acquisition software (ZEISS, 2.6.)<br>Image analysis, phenotyping and single cell isolation: BIAS, Biological Image Analysis Software (ver. 20/12/2020, Single-Cell Technologies Ltd.)<br>Segmentation evaluation: Matlab (9.4.0.813654 (R2018a)).<br>Visualization of microscopy and proteomics results was done using Python 3.7.9., Pandas 1.3.0, Geopandas 0.8.1, Plotly express 0.4.1

For manuscripts utilizing custom algorithms or software that are central to the research but not yet described in published literature, software must be made available to editors and reviewers. We strongly encourage code deposition in a community repository (e.g. GitHub). See the Nature Research guidelines for submitting code & software for further information.

Data

Policy information about availability of data

All manuscripts must include a data availability statement. This statement should provide the following information, where applicable:

- Accession codes, unique identifiers, or web links for publicly available datasets
- A list of figures that have associated raw data
- A description of any restrictions on data availability

The mass spectrometry proteomics data have been deposited to the ProteomeXchange Consortium via the PRIDE partner repository with the dataset identifier PXD023904 (Username: reviewer_pxd023904@ebi.ac.uk, Password: vRAxFOxI).
Numerical source data underlying all graphical representations for Figs. 2b, d, f, g, h; 3c, e, g, h, i, j; 4d,e; 5e, f, g, h, i, j, l and Extended Data Figs. 1a, d, e, f; 2a, b, c, d, e, f; 3a, b, c, d, e, g; 4b, c, d; 5b, c, d, e have been co-submitted with the manuscript as freely accessible files. Primary BIAS imaging data in Figs. 2a,b, e; 3a, d, f; 4a, b, c, f; 5b, c, d, k and Extended Data Figs. 1a,c; 3f; 4a; 5a have been deposited at BioStudies Archive (accession number S-BSST820).There are no restrictions on data availability.

Field-specific reporting

Please select the one below that is the best fit for your research. If you are not sure, read the appropriate sections before making your selection.

☒ Life sciences ☐ Behavioural & social sciences ☐ Ecological, evolutionary & environmental sciences

For a reference copy of the document with all sections, see nature.com/documents/nr-reporting-summary-flat.pdf

Life sciences study design

All studies must disclose on these points even when the disclosure is negative.

Sample size	Sample size calculations were not performed. Experiments were repeated in minimum biological triplicates unless noted otherwise in the corresponding figure legend.
Data exclusions	No collected data were excluded.
Replication	All attempts at replication were successful. Experimental results were confirmed with biological triplicates unless otherwise noted.
Randomization	Randomization of sample groups was performed for mass spectrometric data acquisition. For all other data, no randomization was performed.
Blinding	Investigators were not blinded to sample group allocation during data collection.

Reporting for specific materials, systems and methods

We require information from authors about some types of materials, experimental systems and methods used in many studies. Here, indicate whether each material, system or method listed is relevant to your study. If you are not sure if a list item applies to your research, read the appropriate section before selecting a response.

Materials & experimental systems

n/a	Involved in the study
☐	☒ Antibodies
☐	☒ Eukaryotic cell lines
☒	☐ Palaeontology and archaeology
☒	☐ Animals and other organisms
☐	☒ Human research participants
☒	☐ Clinical data
☒	☐ Dual use research of concern

Methods

n/a	Involved in the study
☒	☐ ChIP-seq
☒	☐ Flow cytometry
☒	☐ MRI-based neuroimaging

Antibodies

Antibodies used

Antibody for immunofluorescence (IF) or immunohistochemistry (IHC):
Rabbit anti-C7orf50 at 1 ug/ml (HPA052281, Atlas Antibodies)
Mouse anti ANLN at 1.25 ug/ml (amab90662, clone CL0303, Atlas Antibodies)
Mouse anti CCNB1 at 1 ug/ml: (610220, BD Biosciences)
EpCAM (Nordic Biosite, Copenhagen K, Denmark, clone BS14, cat. #BSH-7402-1, dilution 1:400)
SOX10; Nordic Biosite, clone BS7 cat. #BSH-7959-1, dilution 1:200
CD146; Cell Marque, Rocklin, CA, USA, clone EP54, cat. #AC-0052, dilution 1:400
Src/c-Src; Cell Signaling Technology, Danvers, Massachusetts, USA, clone 36D10, # 2109, dilution 1:3200

FASN; Cell Signaling Technology, clone C20G5, # 3180, dilution 1:100
CNN1; Cell Marque, Rocklin, CA, USA, clone EP63, #AC-0060, dilution 1:300
CK5; Leica Biosystems, Newcastle Upon Tyne, UK, clone XM26, # NCL-L-CK5, dilution 1:200
FOXJ1 (Mouse, dilution 1:200, 14-9965-80, Invitrogen)
EpCAM (Rabbit, clone D9S3P, dilution 1:200, 14452, Cell Signaling)
Secondary antibody conjugates for IF:
goat anti-rabbit Alexa488 (A11034, Thermo Fisher), goat anti-mouse Alexa555 (A21424, Thermo Fisher), goat anti-chicken Alexa647 (A21449, Thermo Fisher), SYTO 10 for nuclear visualization (10624243, Invitrogen).

Validation

Detailed description about IF antibody validation can be found here:
FOXJ1: https://bit.ly/3wzwOab
EpCAM: https://bit.ly/3NtlUZA
C7orf50: Species reactivity with human samples was verified. The antibody was validated for iIF according to the workflow within the Human Protein Atlas project. See validation information here: https://www.proteinatlas.org/ENSG00000146540-C7orf50/antibody; PMID: 28495876.
ANLN: Species reactivity with human samples was verified. The antibody was validated for IF as part of the Human protein Atlas by staining similarity with an independent antibody. See validation information here: https://www.proteinatlas.org/ENSG00000011426-ANLN/antibody. The antibody has been successfully used in an IF assay in the following article. (PMID: 30103211).
CCNB1: PMID: 29052541
All IHC antibodies are assessed and validated by NordiQC (https://www.nordiqc.org/about.php).
After washing and blocking of endogenous peroxidase activity, the reactions were detected and visualized using Envision FLEX+ High pH kit (Agilent/Dako, # GV800+GV809/GV821) according to the manufacturer's instructions

Eukaryotic cell lines

Policy information about cell lines

Cell line source(s)

The commercial source for the human osteosarcoma cell line U2OS is ATCC (American Type Culture Collection), cell line order number HTB-96. https://www.atcc.org/products/htb-96. U2OS FUCCI cell line was provided by Dr. Sayuri Ito and Dr. Hisao Masai (Tokyo Metropolitan Institute of Medical Science). A clonal U2OS cell line with homogenous and moderate expression levels of Lck-eGFP at the plasma membrane was established from a single colony.

Authentication

All cell lines were authenticated by STR profiling (IdentiCell Molecular Diagnostics).

Mycoplasma contamination

All cell lines were tested negative for mycoplasma (MycoAlert, Lonza).

Commonly misidentified lines
(See ICLAC register)

Cell lines used in this study were not listed in the commonly misidentified category.

Human research participants

Policy information about studies involving human research participants

Population characteristics

not relevant

Recruitment

No patient recruitment was done for the study. Biobanked (FFPE) patient material was used for retrospective studies.

Ethics oversight

We collected archival FFPE tissue samples of salivary gland acinic cell carcinoma and melanoma from the Department of Pathology, Zealand University Hospital, Roskilde, Denmark. Melanoma tissue was from a 51-year-old male and located at the left upper chest. TNM stage at diagnosis was T3aN1M0. Histological subtype was superficial spreading melanoma (SSM), Clark level was 4 and Breslow thickness measured 2.27 mm. Tumor immune infiltration was categorized as non-brisk. The FFPE sample was 17 years old. The patient experienced recurrence at different locations 17 months after diagnosis and died after 71 months. The acinic cell carcinoma was removed from the right parotid gland on a 29-year-old male. There was no sign of mitosis, necrosis dedifferentiation, perineural or intravascular growth. The tumor cells were positive in EpCAM, CK7, DOG1 and SOX10. Mammaglobin was negative. The sample was four years old and the patient is currently disease free. The study was carried out in accordance with the institutional guidelines under approval by the local Medical Ethics Review Committee (SJ-742), the Data Protection Agency (REG-066-2019) and in agreement with Danish law (Medical Research Involving Human Subjects Act). The fallopian tube tissue shown in Fig. 2 is from a 64-years-old female and was macroscopically and histologically normal appearing. All patients have consented before surgery, and patient-derived tissues were obtained fresh or were paraffin embedded according to an approved Institutional Review Board (IRB, 13372B) protocol at the University of Chicago hospital. In accordance with the Medical Ethics Review Committee approval, all FFPE human patient tissue samples were exempted from consent as these studies used existing archived pathological specimens. Human tissue specimens were assessed by a board-certified pathologist.

Note that full information on the approval of the study protocol must also be provided in the manuscript.
